# A pharmacogenetics study of platinum-based chemotherapy in lung cancer: *ABCG2* polymorphism and its genetic interaction with *SLC31A1* are associated with response and survival

**DOI:** 10.7150/jca.51621

**Published:** 2021-01-01

**Authors:** Liyan Wang, Chang Sun, Xiangnan Li, Chenxue Mao, Ji Qian, Jiucun Wang, Junjie Wu, Qiang Li, Chunxue Bai, Baohui Han, Zhiqiang Gao, Jibin Xu, Jiye Yin, Zhaoqian Liu, Daru Lu, Li Jin, Haijian Wang

**Affiliations:** 1 Center for Medical Research and Innovation, Shanghai Pudong Hospital and Pudong Medical Center, Shanghai Medical College, Fudan University, Shanghai, China.; 2 Ministry of Education Key Laboratory of Contemporary Anthropology and Department of Anthropology and Human Genetics, School of Life Sciences, Fudan University, Shanghai, China.; 3 Department of Respiratory and Critical Care Medicine, Changhai Hospital, the Second Military Medical University, Shanghai, China.; 4 Department of Pulmonary Medicine, Zhongshan Hospital of Fudan University, Shanghai, China.; 5 Department of Pneumology, Chest Hospital, Shanghai Jiao Tong University, Shanghai, China.; 6 Department of Cardiothoracic Surgery, Changzheng Hospital of the Second Military Medical University, Shanghai, China.; 7 Department of Clinical Pharmacology, Xiangya Hospital; Hunan Key Laboratory of Pharmacogenomics, Institute of Clinical Pharmacology, Central South University, Changsha, China.

**Keywords:** NSCLC, pharmacogenetics, platinum transporter, ABCG2, SLC31A1, SNP

## Abstract

**Objective:** The expression and function of platinum transporters affect drug tissue concentration and therapeutic effects. We had previously characterized functional variant of platinum intake transporter* SLC31A1* gene. We aimed to investigate the association of platinum efflux transporter gene *ABCG2* polymorphism and combined *ABCG2* and *SLC31A1* polymorphisms with clinical outcomes of NSCLC patients receiving platinum-based chemotherapy.

**Methods:** We genotyped thirteen tagging and functional SNPs of *ABCG2* in 1004 patients, and assessed their association with response, toxicity and survival using unconditional logistic regression and Cox proportional hazards regression analyses respectively.

**Results:** Nonsynonymous rs2231142 (odds ratio [OR] 2.07; 95 % confidence interval [CI] 1.26-3.63), rs1871744 (OR 0.60; 95 % CI 0.42-0.87) and their haplotype and diplotype were associated with objective response. Rs4148157 was associated with shorter overall survival (Log-rank *P* = 0.002; hazard ratio [HR] 1.22; 95 % CI 1.05-1.42). Furthermore, the combined *SLC31A1* rs2233914 and *ABCG2* rs1871744 genotype was significantly associated with poor response (OR 0.31; 95 % CI 0.17-0.56; *P*_interaction_ = 0.003). And the combined genotypes of the functional rs10759637 of *SLC31A1* and the nonsynonymous rs2231142 (Log-rank *P* = 5.20×10^-5^; HR 1.47; 95 % CI 1.19-1.81; *P*_interaction_ = 0.007) or linked rs4148157 of *ABCG2* were significantly associated with poor survival.

**Conclusion:** This study reveals divergent association of *ABCG2* polymorphism with response and survival of NSCLC patients receiving platinum-based chemotherapy, demonstrates the combined effects of functional variants of *ABCG2* and *SLC31A1* on clinical outcomes, and highlights pharmacogenetic relevance of platinum transporter genes interaction.

## Introduction

Lung cancer is one of the most common cancers in both man and woman with high mortality worldwide 1. Non-small cell lung cancer (NSCLC) is the main type (80%) primary lung cancer, and often presents with advanced stage (Ⅲ/Ⅳ) upon first diagnosis 2. The standard treatment for advanced NSCLC is platinum-based combination chemotherapy using cytotoxic compounds such as paclitaxel, navelbine and gemcitabine. However, the response rate is only 20% to 30% and the five-year survival rate is less than 15% 3. Therapeutic response and efficacy are linked to pharmacokinetics and pharmacodynamics pathways including alteration in intracellular drug accumulation mediated by transporters, genetic polymorphisms in these pathways may influence interindividual variability as predictive markers for tailoring chemotherapy with better efficacy and minimal toxicity 3.

Accumulating evidences from studies in cell lines and in clinical setting support reduced drug accumulation as a significant mechanism of platinum resistance 4. The intracellular accumulation of platinum drugs is determined by the plasma-membrane transporters that are responsible for their intake and efflux. Their uptake into cells is mainly mediated by SLC31A1 (solute carrier family 31 member 1), also known as CTR1 (copper transporter 1). Their efflux out of cells is largely mediated by the ATP-binding cassette (ABC) transporters including ABCG2, also known as the breast cancer resistance protein (BCRP), one of most important ABC transporters involved in multidrug resistance of cancer cells. ABCG2 is also expressed in normal tissues including liver, small intestine, colon, kidney and lung, notably in the bronchial epithelium and seromucinous glands, and thus affects the bioavailability and tissue distribution of its substrates 5. In clinical setting of platinum-based chemotherapy for NSCLC, patients with undetectable SLC31A1 in tumors have reduced platinum concentration, decreased tumor response and shorter survival 4, 6, while ABCG2 expression in biopsy specimen predicts shorter survival 7, 8. The aberrant expression and dysfunction of platinum transporters, which are largely ascribed to the functional polymorphisms of their coding genes, may influence interindividual variability in drug tissue concentration and therapeutic effects. We have recently reported that a functional SNP, rs10759637, at the 3′ untranslated region (3′UTR) of *SLC31A1* gene could affect the microRNA-3′UTR interaction, modulate gene expression, and thereby is associated with toxicity and survival of NSCLC patients receiving platinum-based chemotherapy 9. Although the association of *ABCG2* polymorphism with drug resistance has been addressed in a range of solid tumors including lung cancer 5, 10-13, its relevance in outcome prediction for platinum-based chemotherapy of NSCLC is still elusive, and particularly, pharmacogenetic interaction between platinum uptake and efflux transporter genes is largely unknown.

In the present study of NSCLC patients receiving platinum-based treatment (n = 1004), we assessed the association of tagging and functional *ABCG2* SNPs with objective response, survival and toxicities, and also tested the joint effects of *ABCG2* and *SLC31A1* polymorphisms on these clinical outcomes.

## Materials and methods

### Patient recruitment and follow-up

This NSCLC pharmacogenetics study involved two cohorts of patients, the discovery (A) panel (*n* = 237) and the replication (B) panel (*n* = 767). All of the 1004 patients are Chinese Han and were histologically diagnosed with stage Ⅲ-Ⅳ NSCLC between March 2005 and January 2010 from five hospitals in the East of China: Shanghai Chest Hospital, Shanghai Zhongshan Hospital, Shanghai Changhai Hospital, Shanghai Changzheng Hospital, and Cancer Hospital of Jiangsu Province. The recruitment criteria, the demographic and baseline characteristics including gender, age at diagnosis, smoking status, ECOG performance status, TNM stage, and histological type, were described in detail in our previous reports 9, 14-17. The chemotherapeutic regimens were as follows: either cisplatin (75 mg/m^2^) or carboplatin (at an area under the curve 5), both administered on day 1 every 3 weeks, in combination with navelbine (25 mg/m^2^) on days 1 and 8 every 3 weeks, or gemcitabine (1250 mg/m^2^) on days 1 and 8 every 3 weeks, or paclitaxel (175 mg/m^2^) on day 1 every 3 weeks, or docetaxel (75 mg/m^2^) on day 1 every 3 weeks. A few patients received other platinum-based treatment (*n* = 49). Drugs were administered intravenously and treatments lasted for 2 to 6 cycles.

Clinical outcomes including responses, toxicities and survival were assessed. Tumor responses were evaluated after the first two cycles of the course according to the Response Evaluation Criteria in Solid Tumors (RECIST) guidelines version 1.0 18 , which are classified into four categories: complete response (CR), partial response (PR), stable disease (SD), and progressive disease (PD). Toxicity was assessed from the end of the first two cycles of treatment according to the Common Terminology Criteria for Adverse Events version 3.0 (CTCAE v3.0) 19. Progression-free survival (PFS) was calculated from the date of chemotherapy beginning to the date of disease progression or death (whichever occurred first) or the last progression-free follow-up. Overall survival (OS) was calculated from the date of chemotherapy beginning to the date of death. The study was approved by the Ethical Review Committees of Fudan University School of Life Sciences and the participating hospitals, and was conducted in accordance with the Declaration of Helsinki. The written informed consent was obtained from each subject.

### SNPs selection and genotyping

Thirteen tagging and functional SNPs of *ABCG2* were selected. The tagging SNPs were screened from the Han Chinese in Beijing (CHB) population dataset of HapMap phase II database by Haploview 4.1 (http://www.broadinstitute.org/haploview) using a minor allele frequency (MAF) cutoff of 0.05 and a correlation coefficient (*r*^2^) threshold of 0.8. In the setting of common variations and candidate gene based strategy for genetic association study, the linkage disequilibrium (LD)-selected tagSNPs at a relatively stringent *r*^2^ threshold (*r*^2^ > 0.8) resolve >80% of all haplotypes diversity, regardless of recombination, and tag specific haplotypes and clades of related haplotypes in nonrecombinant regions, and analysis of the tagSNP set can comprehensively interrogate for main effects from common functional variation 20. Genomic DNA was extracted from whole blood using the QIAamp DNA Maxi Kit (Qiagen GmbH, Hilden, Germany). Genotyping was performed using iSelect HD BeadChip (Illumina, San Diego, CA, USA).

### Statistical analysis

We used Pearson χ^2^ tests to examine Hardy-Weinberg equilibrium (HWE) for genotype data and test their distribution between groups with various clinical outcomes. We used PHASE version 2.1 to estimate haplotypes 21, used Haploview 4.1 to plot linkage disequilibrium (LD, *D'* and *r*^2^). We measured the association between SNPs and dichotomous clinical phenotypes by calculating odds ratios (OR) and their 95% confidence intervals (CIs) in multivariate unconditional logistic regression analysis, with adjustment of gender, age, smoking status, ECOG performance status, TNM status, histological types and treatment regimen. Haplotype-based association analysis was performed using Haplo.stats package in R-plus (Version: 1.6.8). We tested the null hypotheses of multiplicative gene-gene interactions by evaluating departures from multiplicative joint effect model. Departure from the multiplicative model was assessed by including main effect variables and their product terms in the logistic regression model 22. We analyzed the genetic association with survival by log-rank test with adjustment for covariates. We calculated the hazard ratios (HR) and 95%CI with multivariate Cox proportional hazards regression by adjustment for covariates, and plotted survival curve with Kaplan-Meier method. All statistical analysis was performed by SPSS (version 22). We use the two-side test for all *P* values. A *P* value < 0.05 was considered statistically significant. To account for the issue of multiple testing of SNPs in the Pearson χ^2^ tests of statistical significance of genotypic frequencies of *ABCG2* SNP between response and non-response patients, or between toxicologically graded G0-2 and G3-4 patients, we used SNPSpD to correct the significance threshold taking into account LD between polymorphisms 23. The popular Bonferroni method for multiple test correction may reduce statistical power when the analyzed SNPs show genetic association or in strong LD. Instead, on the basis of the spectral decomposition (SpD) of matrics of pairwise LD between SNPs, the SNPSpD method generates the experiment-wide significance threshold required to keep the Type I error rate at <5%. To account for the issue of multiple testing for log rank comparisons of more than two survival curves in the survival analysis, we used the Bonferroni method for adjustment 24.

## Results

### Patient Characteristics and Clinical Outcomes

The main characteristics for the 1004 eligible patients of the two panel cohorts in total and their clinical outcomes including objective response, toxicity and survival are summarized in Table 1 were documented in detail in our recent report 9. Briefly, 177 (18.1%) of the 975 patients evaluated were responders (one was complete response, CR, and 176 were partial response, PR), and 798 (81.9%) were non-responders (610 showed stable disease, SD, and 188 showed progressive disease, PD). 29.9% of patients evaluated (*n* = 952) showed severe overall toxicity. 8.3% of patients evaluated (*n* = 964) showed severe gastrointestinal toxicity (nausea/vomiting). 23.9% of patients evaluated (*n* = 969) manifested severe hematological toxicity, among which severe thrombocytopenia, neutropenia, leukopenia, anemia were observed in 34 (3.6%), 115 (12.3%), 149 (15.2%) and 29 (3.1%) patients respectively. In the survival analysis, the mortality rate of the cohort patients was 74.9% during a median follow-up of 46.5 months until July 2012 for the final data collection, with median PFS and median OS being 6.5 and 16.0 months respectively. There was no significant difference in the distributions of the demographic and clinical characterizations between the two panels of patients cohorts. In addition, there were comparable rates of objective response and toxicities between our study population and those previously reported in large randomized clinical trials 25.

We selected thirteen tagging and functional SNPs of *ABCG2*, including eleven intronic (rs6857600, rs3109823, rs2725252, rs17731538, rs2231138, rs11931123, rs1871744, rs2231146, rs12505410, rs4148157 and rs2231164) and two nonsynonymous (rs2231137, Val12Met; rs2231142, Gln141Lys) variations, among which the nonsynonymous rs2231142 reportedly leads to reduced ABCG2 protein level and efflux transporter functionality 26. In order to address the consistency of genetic association for the multi-center NSCLC cohorts, we genotyped all of these polymorphisms in both the discovery panel A and the replication panel B. All SNPs are common (MAF > 0.05) in this study population. Their genotypic distributions in panel A (*n* = 237), panel B (*n* = 767) and the combined cohort (*n* = 1004) were all in HWE, and were also comparable with those in the general healthy Chinese Han population in the 1000genome dataset (*n* = 208) (Supplemental Table 1). The LD analysis illustrated relatively strong association between the *ABCG2* polymorphisms in the NSCLC Chinese Han population (Supplemental Figure 1), which is largely consistent with the haplotype architecture of *ABCG2* in Chinese population we previously described by resequencing its exons and regulatory regions 27.

### Association of *ABCG2* polymorphism with response

We compared genotypic distributions of *ABCG2* polymorphisms between responders (CR+PR) and non-responders (SD + PD) (Supplemental Table 2). Rs1871744 consistently showed differential genotypic distribution between responders and non-responders in either panel A (*P* = 0.023), panel B (*P* = 0.042) or the combined cohort (*P* = 0.022). Particularly, the imbalanced distribution of its genotypes, in under-dominant model, that is A/G* vs* A/A+G/G, remained statistically significant after multiple test correction in the combined cohort (*P* = 0.007). Significantly differential genotypic distribution was also observed for rs2231142 in either the total cohort (*P* = 0.005), or panel B (*P* = 0.002), but was not observed in panel A that was much less statistically powered due to its small sample size as compared with panel B. By using logistic regression analysis (Table 2), we found that the A/G genotype of rs1871744, in under-dominant model, was significantly associated with poor response in the total cohort (OR 0.60; 95% CI 0.42-0.87; *P* = 0.006), and this association was of significantly marginal significance in either panel A or panel B. The variant A/A genotype of rs2231142, in recessive model, was significantly associated with favorite response in either the total cohort (OR 2.07; 95% CI 1.26-3.63; *P* = 0.004) or panel B (OR 2.73; 95% CI 1.53-4.85; *P* = 0.001) but not panel A. We also estimated the haplotype and diplotype frequencies for rs1871744 and rs2231142 in the total cohort (Table 3). We predicted three haplotypes in the 975 patients evaluated for response with differential frequencies between responders and non-responders (*P* = 0.046), which was due to overrepresentation in responders of Hap2_AA (composed with their response prone alleles) (*P* = 0.019). Agreeing with the genotype-based association results as above, we consistently observed significant association of favorite response with the Hap2 haplotype (OR 1.45; 95% CI 1.10-1.90; *P* = 0.008) and the Hap2/Hap2 diplotype (OR 2.13; 95% CI 1.28-3.55; *P* = 0.004). Interestingly, in further stratification analysis by demographic and clinical characteristics, the associations of rs1871744 and rs2231142 with response were consistently pronounced in specific subgroups of patients such as men, older than 58, with ECOG PS 0-1, or with squamous cell carcinoma (Supplemental Table 3).

### Joint association of *ABCG2* and *SLC31A1* polymorphisms with response

We have recently reported that *SLC31A1* polymorphisms overall were not associated with response 9, except rs2233914 (G/A), a common variant at 5' flanking region upstream the transcription start site that was associated with poor response in dominant model (OR 0.67; 95% CI 0.48-0.95; *P* = 0.024) (Supplemental Table 4). In an effort to interrogate pharmacogenetically relevant genetic interaction between platinum drug intake and export pathways, we investigated the joint effect of *ABCG2* (rs1871744 and rs2231142) and* SLC31A1* (rs2233914) genotypes on response in the total cohort (Table 4). The combined rs1871744 A/G and rs2233914 (G/A+A/A) group was significantly underrepresented in responders than in non-responders (9.04% *versus* 23.68%; *P* = 2.404×10^-5^, Pearson's Chi-squared test with Yates' continuity correction). Compared to the reference, the presence of only one non-responsive genotype, either of rs1871744 A/G or rs2233914 G/A+A/A were not associated with response. However, their combined group was significantly associated with reduced response opportunity (OR 0.31; 95% CI 0.17-0.56; *P*_interaction_ = 0.003). We did not observe significant joint effect of *SLC31A1* rs2233914 and* ABCG2* and rs2231142 genotypes on response (Supplemental Table 5). In further stratification analysis for the joint effect, the association of combined genotype of rs1871744 A/G and rs2233914 G/A+A/A with poor response was highly significant in male patients (OR 0.20; 95% CI 0.10-0.42), patients older than 58 (OR 0.16; 95% CI 0.06-0.41), patients with ECOG PS 0-1 (OR 0.30; 95% CI 0.16-0.57), smoker patients (OR 0.25; 95% CI 0.11-0.54) and patients with squamous cell carcinoma (OR 0.14; 95% CI 0.04-0.53), which is largely consistent with the stratification spectrum of rs1871744. These results suggest genetic interaction between *SLC31A1* (rs2233914) and* ABCG2* (rs1871744) associated with tumor response to platinum drug chemotherapy.

### Association of *ABCG2* polymorphism with survival

**Figure 1:**
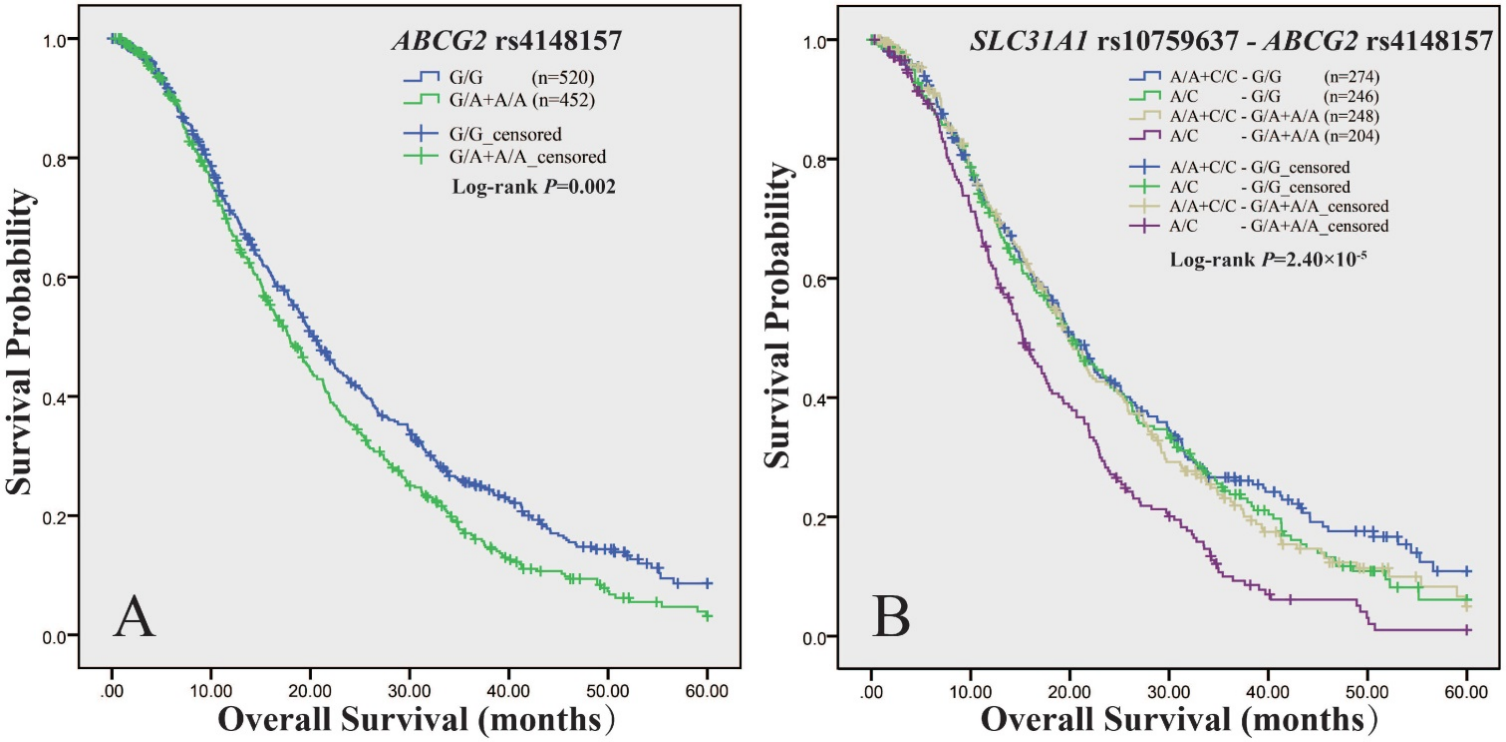
** Kaplan-Meier curve of estimated overall survival for the NSCLC cohort according to *ABCG2* and *SLC31A1* polymorphisms.** The curves were plotted with SPSS software according to the genotypes of *ABCG2* rs4148157 (A), and the combined genotypes of *ABCG2* rs4148157 and* SLC31A1* rs10759637 (B). For *ABCG2* rs4148157 G/A, the G/A+A/A genotypes group was compared to the wild G/G as reference in dominant model. For *SLC31A1*rs10759637 A/C, which had been genotyped in our previous report (Ref 9), the A/C heterozygote was compared to the A/A+C/C homozygotes group as reference in under-dominant model.

We measured association of *ABCG2* polymorphisms with survival in panel A, panel B and the total cohort respectively (Supplemental Table 6). As to PFS, we did not observe any association signal. As to OS, the log-rank test showed that rs4148157, in dominant model, was consistently associated with survival in either panel A (*P* = 0.006), or panel B (*P* = 0.04), or the combined cohort (*P* = 0.002) (Table 5). As shown in Figure 1A, in the total cohort, the median OS time of patients with rs4148157 G/A+A/A was significantly shorter than those with G/G (17.9 *vs* 20.4, *P* = 0.002). Cox proportional hazards regression analysis showed that rs4148157 was associated with disease progression (HR 1.22; 95% CI 1.05-1.42). Notably, in further stratification analysis (Supplemental Table 7), the association of rs4148157 with OS were pronounced in patients with ECOG PS 0-1, patients with IIIB TNM stage, patients with adenocarcinoma, and patients treated with platinum-gemcitabine, respectively. We also observed that rs2231142, which was in strong LD with rs4148157 (*D*′ = 0.94 *r*^2^ = 0.69), was associated with survival in either panel A (log-rank *P* = 0.007) or the total cohort (log-rank *P* = 0.028) but not panel B. These data show that the two linked SNPs of *ABCG2* were associated with survival.

### Joint association of *ABCG2* and *SLC31A1* polymorphisms with survival

We have recently reported that the functional rs10759637 of *SLC31A1* was associated with shorter OS 9. We here further analyzed the joint effect of *SLC31A1* (rs10759637) and* ABCG2* (rs4148157 and rs2231142) on survival in the total cohort (Table 6). The combined genotypes of *SLC31A1* rs10759637 and* ABCG2* rs4148157 were significantly associated with survival (log-rank *P* = 2.50×10^-5^) (Figure 1B). The median OS times of patients with different genotypic combination were 20.4, 20.0, 19.9, and 15.3 months, respectively. The combined group of rs10759637 A/C and rs4148157 G/A+A/A was significantly associated with disease progression (HR 1.58; 95% CI 1.28-1.96). Furthermore, stratification analysis showed that their joint association with shorter OS were significant in male patients (HR 1.54; 95% CI 1.20-1.98), patients older than 58 (HR 1.61; 95% CI 1.19-2.17), patients with ECOG PS 0-1 (HR 1.62; 95% CI 1.30-2.02), smoker patients (HR 1.67; 95% CI 1.27-2.21), patients with IIIB TNM stage (HR 1.75; 95% CI 1.19-2.57), patients with adenocarcinoma (HR 1.49; 95% CI 1.14-1.96), and patients treated with platinum-gemcitabine (HR 2.30; 95% CI 1.42-3.75), respectively. Similarly, joint effect on OS and accordant stratification spectrum with pronounced association signal were also observed for *SLC31A1* (rs10759637) and* ABCG2* (rs2231142) combination (Supplemental Table 8, Supplemental Figure 2). Of note, we also observed significant interactions between *SLC31A1* (rs10759637) and* ABCG2* (rs4148157, *P*_interaction_ = 0.03; rs2231142, *P*_interaction_ = 0.007) associated with OS. These data suggest that genetic interaction between *SLC31A1* and* ABCG2* polymorphisms may be linked with survival outcome.

### *ABCG2* polymorphism and toxicity

We also investigated the effects of *ABCG2* polymorphism on toxicological outcomes. Genotypic distributions between groups with respective mild or severe toxicities are shown in Supplemental Table 9. We found that rs12505410, rs1871744, rs2231142 and rs2231138 displayed differential genotypic distribution, with marginal significance, between groups with mild and severe neutropenia or anemia, and rs12505410 C/C genotype was significantly associated, in recessive model, with neutropenia risk (OR 2.09; 95% CI 1.23-3.57) (Supplemental Table 10).

## Discussion

As the standard treatment for advanced NSCLC, platinum-based chemotherapy achieved varying response and efficacy in patients. The *in situ* expression of plasma-membrane transporters that are responsible for the intake and efflux of platinum drug has been shown to directly affect tissue concentration of drug and thereby be correlated with chemotherapy outcomes 4. In this multi-institutions-based study of NSCLC patients with platinum-based chemotherapy, we characterized divergent association of platinum efflux transporter gene* ABCG2* polymorphism with response and survival, and identified interaction between *ABCG2* and the platinum uptake transporter gene* SLC31A1* associated with clinical outcomes, furthering the pharmacogenetics understanding of platinum-based chemotherapy.

Our result that *ABCG2* nonsynonymous rs2231142 was associated with tumor response to platinum-based chemotherapy is parallel in several lines to both laboratory findings and population observations. Rs2231142 encodes a change from glutamine to lysine at the 141 amino acid in the nucleotide-binding domain of ABCG2. Cell-based studies showed that this missense codon leads to reduced ABCG2 protein level and functionality not only by disrupting protein folding and increasing ubiquitin-mediated lysosomal degradation, but also via enhancing the 3′UTR and microRNA-dependent repression 28-31. Rs2231142 variant reportedly results in decreased substrates efflux in cell lines and affects the pharmacokinetics of, response to, and toxicity of a variety of substrate in clinical settings. For examples, in embryonic kidney cells transfected with *ABCG2*, the variant construct had an 80% higher intracellular concentration of the carcinogenic heterocyclic anime as compared with the wild construct 32. In myelogenous leukemia cell lines, the variant correlated with reduced transport activity, increased cytotoxicity and efficacy after treatment with tyrosine kinase inhibitors 33. Rs2231142 variant genotype has been reported to correlate with reduced *in vivo* intestinal transport capacity 34, high drug plasma in HIV-infected patients treated with dolutegravir 35, much greater plasma area under the concentration-time curve (AUC) values of sulfasalazine, a drug used as ABCG2 probe *in vivo* 36, better low-density lipoprotein cholesterol-lowering efficacy of rosuvastatin in patients with hypercholesterolemia 37, and more favorite response rate of metastatic colorectal cancer to first-line chemotherapy 38. The functional rs2231142 and the intronic rs1871744 are, in genome wide association studies, correlated with serum level of urate, one of endogenous substrates of ABCG2, the susceptibility of gout, which is caused by hyperuricemia, and response to allopurinol in patients with gout 39-41. Consistent with these reports, we observed that the rs2231142 variant genotypes, and its combined diplotype with rs1871744, were associated with favorite response of NSCLC patients to platinum-based chemotherapy. The biological plausibility may be that rs2231142 could decrease ABCG2 expression in overall tissues especially in intestine and lung, increase the bioavailability and the intracellular accumulation of substrate drugs, and thus reinforce their pharmacokinetics properties and pharmacodynamics efficacy.

The functional variant of rs2231142, which is expected to have reduced ABCG2 expression level and capacity, not only correlated with favorite tumor response, as discussed above, but also was reported to be associated with longer survival in the settings of chemotherapy for a few types of cancer 5, 42, 43. However, in contrast to their association with favorite response, we unexpectedly observed that the variant genotypes of rs2231142 and linked rs4148157 of *ABCG2* were associated with shorter survival and disease progression. There are possible explanations for this discrepancy. First, although improved survival is the “gold standard” for evaluating the efficacy of oncologic therapy, and tumor response is a logical surrogate endpoint, since advancing tumor burden is the predominant mechanism by which the disease causes morbidity and mortality, the extent to which tumor response correlates with survival varies 44. Response to chemotherapy could be affected by both a patient's genetic background in genomic DNA and somatic mutation in tumors. During the course of chemotherapy, the selection of increasingly mutated tumor cells will progressively change the tumors genetic makeup from its germ line origin and reduce the impact of original host-specific relevant polymorphism, which could explain why there are some effects on the early tumor response that are not observed again later on survival 10. In accordance with this assumption, we in this study (Supplemental Table 11 and 12) and Muller *et al*. consistently observed that in NSCLC patients receiving first-line chemotherapy, favorable response after the second cycle of the course was significantly correlated with PFS but not with OS 10. Second, the wide ranges of substrates of ABCG2 include not only chemotherapeutic agents such as platinum but also endogenous cell-growth-promoting metabolite molecules such as folates 45, 46. The de-functional ABCG2 rs2231142, on one hand, could maintain accumulation of chemotherapeutic compounds, which would achieve reduced tumor burden. On the other hand, however, it could retain more folic acid, which would lead to high rates of proliferation and worse survival. Indeed, it was recently reported that, for prostate cancer patients with docetaxel treatment, ABCG2 rs2231142 correlates with improved drug response, but also correlates with poorer outcomes possibly through increasing intratumoral folate levels and thereby enhancing tumor cell proliferation 47. Third, because in this study the association of rs4148157 (*P* = 0.008) with shorter survival was much more significant than the marginal signal of the functional rs2231142 (*P* = 0.043), and rs4148157 reportedly has implications for the pharmacokinetics of xenobiotic and endogenous substrates 48, 49, it is plausible that potential *cis-*regulatory function of rs4148157, or its associated causative SNP, may overwhelm the well-established role of rs2231142, and the observed signal for rs2231142 may be due to genetic hitchhiking. Consistent with these scenarios for herein observed divergent associations of *ABCG2* polymorphism with response and survival, similar counter-intuitive results were also reported. In platinum-treated lung cancer patients, carriers with *ABCG2* variant genotype, which are expected to have reduced ABCG2 level, show a shorter OS 10. In patients with head and neck squamous cell carcinoma, high expressions of *ABCB1* and *ABCC1* were associated with favorable survival 50. In patients with childhood neuroblastoma, low (rather than high) *ABCC3* expression were predictive of poor survival 51.

We have previously reported the association of platinum uptake transporter gene *SLC31A1* with clinical outcomes of platinum-based chemotherapy in NSCLC patients, rs10759637 at 3′UTR correlated with shorter OS through reducing SLC31A1 expression, and rs2233914 at 5' flanking region correlated with poor response 9. With an effort to systemically investigate the pharmacogenetic relevance of variations in platinum uptake and efflux transport pathways, we here genotyped the functional and tagging SNPs of *ABCG2* and other five transporter genes (*SLC31A2*, *ATP7A*, *ATP7B*, *ABCB1* and *ABCC2*), but did not observe any significant association signal other than *ABCG2* (data not shown). Consistent with these negative results, it was reported that the expression of ABCB1 in NSCLC cell lines does not correlate with sensitivity to cisplatin or intracellular platinum accumulation, its expression in NSCLC tissues does not correlate with response to cisplatin 4. *Abcc2* knockout in mice does not affect cisplatin disposition and toxicity, *ABCC2* polymorphisms do not correlate with ABCC2 expression and cisplatin-induced cytotoxicity in NCI60 panel and are not associated with cisplatin pharmacokinetics and efficacy in cancer patients 52. In clinical NSCLC specimens, only SLC31A1, but not ATP7A or ATP7B, predicts clinical outcome after platinum-based chemotherapy 53. Of interest, we found that *SLC31A1* rs2233914 and *ABCG2* rs1871744 were jointly associated with response, *SLC31A1* rs10759637 and* ABCG2* rs4148157 were jointly associated with survival. Particularly, the genetic interactions were concomitantly pronounced in subgroups of patients demographically stratified as males, older than 58, ECOG PS 0-1, and smokers. It was reported that reduced *ABCG2* and increased* SLC22A1* mRNA expression are associated with imatinib response in chronic myeloid leukemia 54, and simultaneous high expressions of SLC31A1 and ABCG2 are associated with poor survival of HNSCC patients 50. Cui *et al*. also recently reported the combined effect of* ABCG2* rs2231142 and carboxylesterase 5A gene SNP is associated with platinum-based chemotherapy-induced toxicity in NSCLC patients 55. In a genome-wide association study, additive genetic risk score of nonsynonymous variants of *ABCG2* (rs2231142) and *SLC2A9* (rs16890979) showed graded associations with uric acid and gout 56. It was also recently reported that the association of *ABCG2* rs2231142 with hyperuricemia is modified by *SLC2A9* polymorphism in an elderly Chinese population 57. Although the causative mechanism for the synergistic effects of polymorphisms in platinum intake and export pathways, such as *SLC31A1* and *ABCG2*, is still largely unknown, the association of their interaction with clinical outcomes suggests the combined relevant genotypes of *SLC31A1* and *ABCG2* as potential pertinent and actionable pharmacogenetic biomarkers for platinum-based chemotherapy of NSCLC, especially for some demographically stratified subgroups patients.

Notably, the functional polymorphisms of *SLC31A1* and *ABCG2* genes have highly variable frequencies depending on ethnicity. In public SNP database, the low-expression-related ancestral allele (C) of *SLC31A1* rs10759637 is common in African populations, while the high-expression-related derived allele (A) is dominant in Caucasian and Chinese Han population. The low-function-related derived allele (A) of *ABCG2* rs2231142 is prevalent in Eastern Asian populations (25-35%), common in Caucasian (8-15%) but rare in Sub-Saharan (0.9%) and African American (0-5%). The pronounced overrepresentation of the high-expression allele of uptake transporter *SLC31A1* and the low-function allele of efflux transporter *ABCG2* in non-Africans, as compared with in Africans, strongly suggest divergent pharmacokinetics of platinating agents between them. Indeed, *in vitro* study showed that B-lymphoblastoid cell lines derived from Caucasians of the HapMap project are more susceptible to cytotoxicity induced by carboplatin than those cells from Africans 58. In a cohort of NSCLC patients with neoadjuvant platinum-based chemotherapy, Kim *et al*. reported that the African American had significantly reduced SLC31A1 expression in tumor, lowered tissue platinum concentration and decreased tumor shrinkage as compared to Caucasians 6. Thus, our findings that the genetic interaction between *SLC31A1* and *ABCG2* polymorphisms was associated with clinical outcomes of NSCLC patients receiving platinum-based chemotherapy may have important implications for the pharmacoethnicity of platinating agents in cancer chemotherapy.

We should acknowledge that this work may have some limitations. All of the 1004 patients are Chinese Han and were recruited from five hospitals in East China. In order to address the replicability of genetic association, we grouped this cohort into the discovery (A) panel (*n* = 237) and the replication (B) panel (*n* = 767) according to their institutional and regional sources, and genotyped all of the selected SNPs of *ABCG2* in the total cohort. There was no significant difference in the characterized demographic and clinical features for the two panels. Furthermore, the observed genotypic frequencies of *ABCG2* polymorphisms in either panel A, panel B and the total cohort all fit well with the Hardy-Weinberg law, and are also much comparable to those in the general healthy Chinese Han population in the 1000 genome dataset. Our previous genomic dissection of population substructure of Chinese Han also indicates that there is no apparent population differentiation in East Chinese Han, the general natural population in this study, from the one-dimensional “north-south” structure 59. It is thus at least possible that the association of *ABCG2* polymorphisms with clinical outcomes of NSCLC patients receiving platinum-based chemotherapy could be due to sampling and ascertainment biases and population stratification. We here emphasized rs1871744 and rs4148157 because only the two SNPs were consistently associated with response and survival respectively in both panel A, panel B and the total cohort. However, we still found differential and even divergent association signals among the three populations for other *ABCG2* SNPs including the nonsynonymous rs2231142, which was associated with response in panel B and with survival in panel A respectively. Therefore, further validations of the findings of this work in cohorts of different ethnic populations with larger sample size are highly warranted.

In summary, this pharmacogenetic study demonstrates that platinum efflux transporter gene* ABCG2* polymorphism was divergently associated with objective response and overall survival of NSCLC patients receiving platinum-based chemotherapy, and its interaction with the platinum import transporter gene* SLC31A1* polymorphism was also associated with clinical outcomes. These findings may provide potential predictive markers for clinical outcomes of platinum-based chemotherapy of NSCLC and have implications for pharmacogenetics of platinating agents based cancer chemotherapy.

## Supplementary Material

Supplementary figures and tables.

## Figures and Tables

**Table 1:** Patient characteristics and clinical outcomes (n = 1004)

Characteristic	Panel A		Panel B		*P* value *^d^*	All	
	Total Number	n (%)	Total Number	n (%)		Total Number	n (%)
All patients	237		767			1004	
**Sex**	237		767			1004	
Male		175 (73.8)		531 (69.2)	0.202		706 (70.3)
Female		62 (26.2)		236 (30.8)			298 (29.7)
**Median age (yrs)**	237	59	767	58		1004	58
≤ Median age		123 (51.9)		404 (52.7)	0.893		518 (51.6)
> Median age		114 (48.1)		363 (47.3)			486 (48.4)
**Smoking Status**	237		763			1000	
Ever Smoker		140 (59.1)		435 (56.6)	0.628		575 (57.5)
Nonsmoker*^a^*		97 (40.9)		328 (43.4)			425 (42.5)
**ECOG performance status*^b^***	234		756			990	
0-1		217 (92.7)		687 (90.9)	0.453		904 (91.3)
2		17 (7.3)		69 (9.1)			86 (8.7)
**TNM stage**	226		773			999	
IIIA		19 (8.0)		62 (8.1)	0.073		81 (8.1)
IIIB		83 (35.2)		210 (27.5)			293 (29.3)
IV		134 (56.8)		491 (64.4)			625 (62.6)
**Histological type**	237		767			1004	
Adenocarcinoma (AC)		143 (60.3)		489 (63.8)	0.533		632 (62.9)
Squamous cell carcinoma (SCC)		53 (22.4)		168 (21.9)			221 (22.1)
Adenosquamocarcinoma		7 (2.9)		13 (1.7)			20 (2.0)
Others*^c^*		34 (14.4)		97 (12.6)			131 (13.0)
**Chemotherapy regimens**	237		767			1004	
Platinum (cisplatin)-navelbine		84 (35.4)		232 (30.2)	0.110		316 (31.5)
Platinum (cisplatin)-gemcitabine		52 (21.9)		187 (24.4)			239 (23.8)
Platinum (carboplatin)-paclitaxel		61 (25.7)		252 (32.9)			313 (31.2)
Platinum-docetaxel		25 (10.5)		62 (8.1)			87 (8.7)
Other platinum combinations		15 (6.5)		34 (4.4)			49 (4.9)
**Objective response**	234		741			975	
Complete response (CR)		1 (0.4)		0 (0.0)	0.106		1 (0.1)
Partial response (PR)		43 (18.4)		133 (17.9)			176 (18.0)
Stable disease (SD)		154 (65.8)		456 (61.5)			610 (62.6)
Progressive disease (PD)		36 (15.4)		152 (20.6)			188 (19.3)
Toxicity outcome							
**Grade 3 or 4 gastrointestinal toxicity**							
Nausea/vomiting	225	16 (7.1)	739	64 (8.7)	0.587	964	80 (8.3)
Grade 3 or 4 hematologic toxicity	223	52 (23.3)	746	180 (24.1)	0.914	969	232 (23.9)
Anemia	223	12 (5.4)	721	17 (2.4)	0.048	944	29 (3.1)
Leukopenia	227	34 (15.0)	753	115 (15.3)	1.000	980	149 (15.2)
Neutropenia	217	19 (8.8)	718	96 (13.4)	0.133	935	115 (12.3)
Thrombocytopenia	226	9 (4.0)	724	25 (3.5)	0.876	950	34 (3.6)
Grade 3 or 4 overall toxicity	222	60 (27.0)	730	225 (30.8)	0.472	952	285 (29.9)
**Median time to outcomes (months)**	228		744			972	
Progression-free survival (PFS)		6.6		6.5			6.5
Overall survival (OS)		17.5		15.7			16.0

*^a^* Nonsmokers were defined as those who had smoked <1 cigarette per day and for <1 year in their lifetime. *^b^*ECOG PS, Eastern Cooperative Oncology Group performance status. *^c^* Other carcinomas included mixed cell or undifferentiated carcinoma. *^d^ P* values of Pearson χ^2^ tests for differences between panel A and B.

**Table 2:** Association between *ABCG2* SNPs and objective response

Genotype	Panel A			Panel B			All		
	Response (CR+PR/SD+PD)	*P* value*^a^*	OR (95%CI)*^b^*	Response (CR+PR/SD+PD)	*P* value *^a^*	OR (95%CI)*^b^*	Response (CR+PR/SD+PD)	*P* value*^a^*	OR (95%CI)*^b^*
rs2231164									
A/A	16/51	0.274	1.00 (ref)	33/173	0.028	1.00 (ref)	49/224	0.035	1.00 (ref)
A/G	16/92		0.54 (0.23-1.26)	60/316		1.09(0.68-1.77)	76/408		0.89 (0.59-1.34)
G/G	12/47		0.77 (0.31-1.95)	40/119		**1.94 (1.13-3.32)**	52/166		1.47 (0.93-2.32)
G/G *vs* A/A+A/G	32/143	0.528	1.12 (0.51-2.46)	93/489	0.008	**1.83 (1.17-2.84)**	125/632	0.013	**1.58 (1.08-2.32)**
rs4148157									
G/G	23/100	0.433	1.00 (ref)	63/335	0.036	1.00 (ref)	86/435	0.043	1.00 (ref)
G/A	16/75		0.69 (0.32-1.53)	57/245		1.35 (0.90-2.04)	73/320		1.17 (0.82-1.68)
A/A	5/15		1.86 (0.57-6.05)	13/28		**2.65(1.23-5.70)**	18/43		**2.12 (1.13-4.00)**
A/A *vs* G/G+G/A	39/175	0.458	2.19 (0.71-6.75)	120/580	0.018	**2.31 (1.11-4.84**)	159/755	0.017	**2.00 (1.08-3.71)**
rs1871744									
A/A	25/98	0.023	1.00 (ref)	77/279	0.042	1.00 (ref)	102/377	0.022	1.00 (ref)
A/G	9/72		0.51 (0.21-1.23)	44/254		**0.59 (0.39-0.91)**	53/326		**0.59 (0.41-0.86)**
G/G	10/20		2.35 (0.87-6.31)	12/75		0.59 (0.30-1.18)	22/95		0.92 (0.54-1.58)
A/G* vs* A/A+G/G	35/118	0.022	**0.43 (0.18-0.99)**	91/362	0.064	**0.65 (0.43-0.98)**	124/472	0.007 *^c^*	**0.60 (0.42-0.87)**
rs2231142									
C/C	21/89	0.618	1.00 (ref)	55/292	0.002	1.00 (ref)	76/381	0.005 *^c^*	1.00 (ref)
C/A	17/80		0.83 (0.39-1.78)	55/270		1.17 (0.76-1.79)	72/350		1.07 (0.74-1.54)
A/A	6/21		1.13 (0.37-3.43)	23/46		**2.96 (1.59-5.45)**	29/67		**2.14 (1.26-3.63)**
A/A *vs* C/C+C/A	38/169	0.348	1.24 (0.44-3.51)	110/562	4.71×10^-4^	**2.73 (1.53-4.85)**	148/731	0.001 *^c^*	**2.07 (1.26-3.63)**

*^a^ P* values of Pearson χ^2^ tests. *^b^* Odds ratios (OR) and their 95% confidence intervals (CIs) and *P* values were calculated with unconditional logistic regression analysis, with adjustment of gender, age, smoking status, ECOG performance status, TNM status, histological types, and treatment regimen. *^c^* Statistical significance remained after multiple tests adjustment taking into account linkage disequilibrium between polymorphisms.

**Table 3:** Association between *ABCG2* haplotype and diplotype and objective response

Haplotype or Diplotype*^a^*	Response (CR+PR/SD+PD)	*P*-value*^b^*	OR (95% CI)*^c^*	*P*-value*^c^*
**Haplotype frequency**				
Hap1_AC	127/596	0.605	1.00	
Hap2_AA	130/484	**0.019**	**1.45 (1.10-1.90)**	**0.008**
Hap3_GC	97/516	0.071	1.08 (0.62-1.88)	0.781
**Diplotype frequency**				
Non-Hap2 carriers	104/530	**0.005**	1.00	
Hap2-Others carriers	44/201		1.10 (0.73-1.65)	0.643
Hap2-Hap2 carriers	29/67		**2.13 (1.28-3.55)**	**0.004**

*^a^* Haplotypes were predicted with PHASE basing on rs1871744(A/G) and rs2231142 (C/A) that were associated with ORR outcomes *^b^ P*-values of Pearson χ^2^ test for the difference in haplotype and diplotype frequencies. *^c^* Odds ratios (OR) and their 95% confidence intervals (CIs) and *P* values were calculated with unconditional logistic regression analysis, with adjustment of gender, age, smoking status, ECOG performance status, TNM status, histological types, and treatment regimen.

**Table 4:** Joint association of* SLC31A1* rs2233914 (G/A) and *ABCG2* rs1871744 (A/G) with objective response

Stratification subgroup	Genotype (*SLC31A1*—*ABCG2*)*^a^*	Response (CR+PR/SD+PD)	OR (95% CI)*^b^*	*P* value*^b^*
All	[G/G] - [A/A+G/G]	58/215	1.00 (ref)	
	[A/G+A/A] - [A/A+G/G]	66/257	0.93 (0.62-1.41)	0.740
	[G/G] - [A/G]	37/137	0.96 (0.59-1.57)	0.875
	[A/G+A/A] - [A/G]	16/189	**0.31 (0.17-0.56)***^c^*	**1.23×10^-4^**
**Gender**				
Male	[G/G] - [A/A+G/G]	49/141	1.00 (ref)	
	[A/G+A/A] - [A/A+G/G]	50/189	0.73 (0.45-1.17)	0.190
	[G/G] - [A/G]	22/94	0.56 (0.31-1.04)	0.066
	[A/G+A/A] - [A/G]	10/134	**0.20 (0.10-0.42)**	**2.50×10^-5^**
Female	[G/G] - [A/A+G/G]	9/74	1.00 (ref)	
	[A/G+A/A] - [A/A+G/G]	16/68	1.65 (0.64-4.24)	0.299
	[G/G] - [A/G]	15/43	**3.19 (1.22-8.33)**	**0.018**
	[A/G+A/A] - [A/G]	6/55	0.85 (0.27-2.67)	0.778
**Age**				
≤58	[G/G] - [A/A+G/G]	23/126	1.00 (ref)	
	[A/G+A/A] - [A/A+G/G]	28/127	1.12 (0.60-2.11)	0.719
	[G/G] - [A/G]	22/70	1.51 (0.76-3.03)	0.243
	[A/G+A/A] - [A/G]	10/96	0.54 (0.24-1.23)	0.141
>58	[G/G] - [A/A+G/G]	35/89	1.00 (ref)	
	[A/G+A/A] - [A/A+G/G]	38/130	0.69 (0.39-1.23)	0.211
	[G/G] - [A/G]	15/67	0.53 (0.26-1.11)	0.091
	[A/G+A/A] - [A/G]	6/93	**0.16 (0.06-0.41)**	**1.46×10^-4^**
**ECOG PS**				
0-1	[G/G] - [A/A+G/G]	53/199	1.00 (ref)	
	[A/G+A/A] - [A/A+G/G]	64/223	1.02 (0.66-1.58)	0.922
	[G/G] - [A/G]	31/123	0.95 (0.57-1.60)	0.849
	[A/G+A/A] - [A/G]	14/171	**0.30 (0.16-0.57)**	**2.44×10^-4^**
2	[G/G] - [A/A+G/G]	5/12	1.00 (ref)	
	[A/G+A/A] - [A/A+G/G]	2/29	**0.04 (0.00-0.53)**	**0.014**
	[G/G] - [A/G]	5/12	0.47 (0.04-5.36)	0.546
	[A/G+A/A] - [A/G]	2/17	0.18 (0.01-2.98)	0.230
**Smoking status**				
Nonsmoker	[G/G] - [A/A+G/G]	20/103	1.00 (ref)	
	[A/G+A/A] - [A/A+G/G]	22/100	1.15 (0.57-2.32)	0.703
	[G/G] - [A/G]	21/57	2.06 (1.00-4.28)	0.052
	[A/G+A/A] - [A/G]	6/82	0.40 (0.15-1.08)	0.070
Smoker	[G/G] - [A/A+G/G]	38/112	1.00 (ref)	
	[A/G+A/A] - [A/A+G/G]	44/156	0.80 (0.47-1.36)	0.409
	[G/G] - [A/G]	16/80	0.52 (0.26-1.06)	0.072
	[A/G+A/A] - [A/G]	10/107	**0.25 (0.11-0.54)**	**4.27×10^-4^**
**TNM stage**				
IIIA	[G/G] - [A/A+G/G]	9/12	1.00 (ref)	
	[A/G+A/A] - [A/A+G/G]	9/17	0.47 (0.13-1.80)	0.272
	[G/G] - [A/G]	6/10	0.53 (0.11-2.62)	0.436
	[A/G+A/A] - [A/G]	3/13	0.24 (0.05-1.24)	0.088
IIIB	[G/G] - [A/A+G/G]	19/72	1.00 (ref)	
	[A/G+A/A] - [A/A+G/G]	19/63	1.20 (0.56-2.60)	0.637
	[G/G] - [A/G]	15/32	1.66 (0.70-3.93)	0.247
	[A/G+A/A] - [A/G]	3/61	**0.18 (0.05-0.66)**	**0.010**
IV	[G/G] - [A/A+G/G]	30/130	1.00 (ref)	
	[A/G+A/A] - [A/A+G/G]	38/175	0.86 (0.50-1.50)	0.604
	[G/G] - [A/G]	16/93	0.76 (0.38-1.50)	0.423
	[A/G+A/A] - [A/G]	10/115	**0.38 (0.17-0.82)**	**0.013**
**Histological type**				
AC	[G/G] - [A/A+G/G]	24/146	1.00 (ref)	
	[A/G+A/A] - [A/A+G/G]	24/173	0.84 (0.45-1.56)	0.577
	[G/G] - [A/G]	19/84	1.34 (0.68-2.64)	0.391
	[A/G+A/A] - [A/G]	11/131	0.50 (0.23-1.07)	0.072
SCC	[G/G] - [A/A+G/G]	20/37	1.00 (ref)	
	[A/G+A/A] - [A/A+G/G]	24/46	0.99 (0.46-2.12)	0.969
	[G/G] - [A/G]	15/38	0.72 (0.30-1.70)	0.451
	[A/G+A/A] - [A/G]	3/34	**0.14 (0.04-0.53)**	**0.004**
**Therapy regimens**				
Pt-navelbine	[G/G] - [A/A+G/G]	21/62	1.00 (ref)	
	[A/G+A/A] - [A/A+G/G]	28/78	0.97 (0.49-1.94)	0.936
	[G/G] - [A/G]	17/36	1.41 (0.63-3.15)	0.406
	[A/G+A/A] - [A/G]	7/53	**0.33 (0.13-0.87)**	**0.025**
Pt-gemcitabine	[G/G] - [A/A+G/G]	13/54	1.00 (ref)	
	[A/G+A/A] - [A/A+G/G]	15/61	0.90 (0.38-2.17)	0.820
	[G/G] - [A/G]	4/29	0.52 (0.14-1.89)	0.323
	[A/G+A/A] - [A/G]	5/50	0.47 (0.15-1.47)	0.195
Pt-paclitaxe	[G/G] - [A/A+G/G]	20/72	1.00 (ref)	
	[A/G+A/A] - [A/A+G/G]	13/77	0.59 (0.25-1.38)	0.223
	[G/G] - [A/G]	13/54	0.89 (0.38-2.08)	0.786
	[A/G+A/A] - [A/G]	3/58	**0.19 (0.05-0.70)**	**0.012**
Pt-docetaxel	[G/G] - [A/A+G/G]	3/15	1.00 (ref)	
	[A/G+A/A] - [A/A+G/G]	4/29	1.54 (0.16-15.34)	0.711
	[G/G] - [A/G]	3/10	0.55 (0.06-5.16)	0.597
	[A/G+A/A] - [A/G]	1/20	0.25 (0.01-4.70)	0.352

*^a^ SLC31A1* rs2233914 (G/A) for the study subjects had been genotyped in our previous report (Ref 9). *^b^* Odds ratios (OR) and their 95% confidence intervals (CIs) and *P* values were calculated with unconditional logistic regression analysis, with adjustment of gender, age, smoking status, ECOG performance status, TNM status, histological types, and treatment regimen. *^c^* Test of interaction for the cohort of all patients with *P* value being 0.003.

**Table 5:** Association between *ABCG2* SNPs and overall survival

Genotype	Panel A				Panel B				All			
	n/N*^a^*	MST (m)*^b^*	Log-rank* P*	HR (95% CI)*^c^*	n/N*^a^*	MST (m)*^b^*	Log-rank* P*	HR (95% CI)*^c^*	n/N*^a^*	MST (m)*^b^*	Log-rank* P*	HR (95% CI)*^c^*
**rs2231164**												
A/A	47/65	22.4	**0.025**	1.00 (ref)	152/205	19.1	0.383	1.00 (ref)	199/270	20.7	**0.039**	1.00 (ref)
A/G	79/104	19.3		1.36 (0.92-2.00)	276/379	18.8		1.01 (0.83-1.24)	355/483	19.0		1.08 (0.91-1.29)
G/G	47/59	15.8		**1.86 (1.18-2.94)**	127/160	18.2		1.15 (0.91-1.47)	174/219	18.0		**1.26 (1.02-1.55)**
**rs4148157**												
G/G	84/118	21.8	**0.018**	1.00 (ref)	287/402	19.0	0.120	1.00 (ref)	371/520	20.4	**0.008*^d^***	1.00 (ref)
G/A	72/90	15.6		1.35 (0.95-1.92)	236/300	18.9		**1.20 (1.00-1.43)**	308/390	17.9		**1.21 (1.04-1.42)**
A/A	17/0	15.1		1.67 (0.95-2.93)	32/42	20.2		1.14 (0.78-1.66)	49/62	17.0		1.25 (0.92-1.70)
G/A+A/A* vs* G/G	89/110	15.4	**0.006**	**1.40 (1.01-1.96)**	268/342	18.9	**0.040**	**1.19 (1.01-1.41)**	357/452	17.9	**0.002**	**1.22 (1.05-1.42)**
**rs12505410**												
A/A	92/114	18.7	0.083	1.00 (ref)	245/327	17.0	0.350	1.00 (ref)	337/441	17.1	0.064	1.00 (ref)
A/C	60/85	21.4		0.73 (0.51-1.04)	241/327	19.7		0.85 (0.71-1.03)	301/412	20.7		**0.84 (0.72-0.99)**
C/C	21/29	21.5		0.82 (0.49-1.36)	69/90	22.2		0.78 (0.59-1.03)	90/119	21.6		**0.77 (0.61-0.96)**
**rs2231142**												
C/C	76/106	21.7	**0.025**	1.00 (ref)	248/327	19.0	0.326	1.00 (ref)	327/452	20.4	**0.043**	1.00 (ref)
C/A	73/95	15.4		**1.49 (1.05-2.10)**	248/329	18.9		1.06 (0.88-1.27)	321/422	17.9		1.14 (0.97-1.33)
A/A	24/27	21.2		1.46 (0.88-2.42)	60/76	16.5		1.21 (0.90-1.63)	80/98	19.6		1.27 (0.99-1.64)
C/A+A/A *vs* C/C	97/122	16.6	**0.007**	**1.48 (1.07-2.06)**	308/405	18.9	0.336	1.08 (0.91-1.29)	401/520	18.0	**0.028**	1.16 (1.00-1.35)

*^a^* Numbers indicate the death event for NSCLC patients during the following-up time among all individuals in the same genotype group. *^b^*MST: median survival time. *^c^*Hazard ratios (HR) and their 95% confidence intervals (CIs) and *P* values were calculated with by multivariate Cox proportional hazards regression with adjustment for covariates. *^d^* Statistical significance remained after Bonferroni multiple tests.

**Table 6:** Joint association of* SLC31A1* rs10759637 (A/C) and* ABCG2* rs4148157 (G/A) with overall survival

Stratification subgroup	Genotype (*SLC31A1*—*ABCG2*)*^a^*	n/N*^b^*	MST (m)*^c^*	Log-rank* P*	HR (95% CI)*^d^*	*P* value*^d^*
All	[A/A+C/C] - [G/G]	188/274	20.4	**2.50×10^-5 *f*^**	1.00 (ref)	
	[A/C] - [G/G]	183/246	20.0		1.08 (0.87-1.33)	0.482
	[A/A+C/C] - [G/A+A/A]	184/248	19.9		1.07 (0.86-1.31)	0.557
	[A/C] - [G/A+A/A]	173/204	15.3		**1.58 (1.28-1.96)***^e^*	**2.40×10^-5^**
**Gender**						
Male	[A/A+C/C] - [G/G]	143/196	19.5	**0.002^*f*^**	1.00 (ref)	
	[A/C] - [G/G]	123/168	19.1		1.04 (0.81-1.33)	0.752
	[A/A+C/C] - [G/A+A/A]	138/184	19.2		1.02 (0.80-1.30)	0.888
	[A/C] - [G/A+A/A]	124/141	14.9		**1.54 (1.20-1.98)**	**0.001**
Female	[A/A+C/C] - [G/G]	45/78	28.8	**0.013**	1.00 (ref)	
	[A/C] - [G/G]	60/78	23.8		1.18 (0.78-1.79)	0.428
	[A/A+C/C] - [G/A+A/A]	46/64	21.4		1.30 (0.84-2.03)	0.243
	[A/C] - [G/A+A/A]	49/63	16.8		**1.73 (1.12-2.66)**	**0.013**
**Age**						
≤58	[A/A+C/C] - [G/G]	103/157	22.3	**0.010**	1.00 (ref)	
	[A/C] - [G/G]	86/126	22.7		0.98 (0.73-1.32)	0.896
	[A/A+C/C] - [G/A+A/A]	94/128	21.2		1.09 (0.81-1.46)	0.572
	[A/C] - [G/A+A/A]	76/94	17.9		**1.51 (1.11-2.06)**	**0.009**
>58	[A/A+C/C] - [G/G]	85/117	18.8	**0.004*^f^***	1.00 (ref)	
	[A/C] - [G/G]	97/120	17.7		1.11 (0.82-1.51)	0.485
	[A/A+C/C] - [G/A+A/A]	90/120	19.3		0.96 (0.70-1.31)	0.788
	[A/C] - [G/A+A/A]	97/110	14.3		**1.61 (1.19-2.17)**	**0.002**
**ECOG PS**						
0-1	[A/A+C/C] - [G/G]	172/253	21.0	**3.00×10^-6*f*^**	1.00 (ref)	
	[A/C] - [G/G]	156/215	20.9		1.06 (0.85-1.32)	0.604
	[A/A+C/C] - [G/A+A/A]	168/223	20.0		1.08 (0.87-1.34)	0.491
	[A/C] - [G/A+A/A]	160/188	15.2		**1.62 (1.30-2.02)**	**1.60×10^-5^**
2	[A/A+C/C] - [G/G]	12/16	17.8	0.800	1.00 (ref)	
	[A/C] - [G/G]	23/27	12.4		0.82 (0.34-1.97)	0.658
	[A/A+C/C] - [G/A+A/A]	15/24	19.1		0.43 (0.16-1.14)	0.089
	[A/C] - [G/A+A/A]	11/13	21.4		0.67 (0.24-1.88)	0.444
**Smoking status**						
Nonsmoker	[A/A+C/C] - [G/G]	72/116	22.4	**0.042**	1.00 (ref)	
	[A/C] - [G/G]	76/101	23.8		1.03 (0.73-1.44)	0.870
	[A/A+C/C] - [G/A+A/A]	75/101	20.3		1.04(0.73-1.46)	0.843
	[A/C] - [G/A+A/A]	68/87	17.2		**1.42 (1.00-2.00)**	**0.048**
Smoker	[A/A+C/C] - [G/G]	116/158	20.2	**0.001*^ f^***	1.00 (ref)	
	[A/C] - [G/G]	106/144	18.1		1.10 (0.84-1.45)	0.483
	[A/A+C/C] - [G/A+A/A]	108/146	19.2		1.07 (0.82-1.40)	0.624
	[A/C] - [G/A+A/A]	103/115	14.3		**1.67(1.27-2.21)**	**2.99×10^-4^**
**TNM stage**						
IIIA	[A/A+C/C] - [G/G]	14/20	31.3	0.192	1.00 (ref)	
	[A/C] - [G/G]	15/21	15.3		**2.54 (1.04-6.20)**	**0.041**
	[A/A+C/C] - [G/A+A/A]	13/21	25.5		**3.20 (1.28-8.00)**	**0.013**
	[A/C] - [G/A+A/A]	14/14	16.5		**2.81 (1.06-7.47)**	**0.038**
IIIB	[A/A+C/C] - [G/G]	64/89	21.0	**0.004*^ f^***	1.00 (ref)	
	[A/C] - [G/G]	43/57	22.5		0.99 (0.66-1.50)	0.976
	[A/A+C/C] - [G/A+A/A]	55/75	17.0		1.37 (0.95-1.99)	0.095
	[A/C] - [G/A+A/A]	53/62	15.1		**1.75 (1.19-2.57)**	**0.004**
IV	[A/A+C/C] - [G/G]	110/165	18.3	**0.019**	1.00 (ref)	
	[A/C] - [G/G]	124/167	19.6		1.02 (0.79-1.33)	0.875
	[A/A+C/C] - [G/A+A/A]	115/150	21.3		0.93 (0.71-1.22)	0.604
	[A/C] - [G/A+A/A]	104/126	15.6		**1.42 (1.08-1.87)**	**0.013**
**Histological type**						
AC	[A/A+C/C] - [G/G]	117/175	21.8	**0.004*^ f^***	1.00 (ref)	
	[A/C] - [G/G]	119/158	22.5		1.02 (0.78-1.32)	0.900
	[A/A+C/C] - [G/A+A/A]	113/148	19.6		1.18 (0.91-1.54)	0.214
	[A/C] - [G/A+A/A]	105/131	16.5		**1.49 (1.14-1.96)**	**0.004**
SCC	[A/A+C/C] - [G/G]	48/66	19.3	**0.044**	1.00 (ref)	
	[A/C] - [G/G]	34/50	13.1		1.26 (0.79-2.01)	0.334
	[A/A+C/C] - [G/A+A/A]	37/54	21.9		0.75 (0.47-1.19)	0.218
	[A/C] - [G/A+A/A]	42/43	14.3		1.48 (0.93-2.33)	0.096
**Therapy regimens**						
Pt-navelbine	[A/A+C/C] - [G/G]	61/90	21.8	0.178	1.00 (ref)	
	[A/C] - [G/G]	55/72	23.3		1.04 (0.71-1.52)	0.837
	[A/A+C/C] - [G/A+A/A]	63/84	21.3		0.89 (0.62-1.28)	0.532
	[A/C] - [G/A+A/A]	50/60	16.1		**1.48 (1.01-2.16)**	**0.045**
Pt-gemcitabine	[A/A+C/C] - [G/G]	37/62	22.5	**0.002*^f^***	1.00 (ref)	
	[A/C] - [G/G]	52/68	20.9		1.24 (0.80-1.92)	0.343
	[A/A+C/C] - [G/A+A/A]	43/57	19.1		1.50 (0.95-2.39)	0.084
	[A/C] - [G/A+A/A]	42/49	13.3		**2.30 (1.42-3.75)**	**0.001**
Pt-paclitaxe	[A/A+C/C] - [G/G]	62/79	18.2	0.112	1.00 (ref)	
	[A/C] - [G/G]	53/76	19.1		0.88 (0.60-1.29)	0.511
	[A/A+C/C] - [G/A+A/A]	58/80	20.0		0.80 (0.55-1.15)	0.232
	[A/C] - [G/A+A/A]	55/65	15.3		1.14 (0.78-1.67)	0.494
Pt-docetaxel	[A/A+C/C] - [G/G]	20/29	20.4	0.527	1.00 (ref)	
	[A/C] - [G/G]	17/22	15.2		2.14 (0.88-5.22)	0.093
	[A/A+C/C] - [G/A+A/A]	10/14	17.0		0.91 (0.41-2.06)	0.826
	[A/C] - [G/A+A/A]	17/18	17.4		1.78 (0.86-3.71)	0.121

*^a^ SLC31A1* rs2233914 (G/A) for the study subjects had been genotyped in our previous report (Ref 9). *^b^*Numbers indicate the death event for NSCLC patients during the following-up time among all individuals in the same genotype group. *^c^*MST: median survival time. *^d^*Hazard ratios (HR) and their 95% confidence intervals (CIs) and *P* values were calculated with by multivariate Cox proportional hazards regression with adjustment for covariates. *^e^*Test of interaction for the cohort of all patients with *P* value being 0.030. *^f^* Statistical significance remained after Bonferroni multiple tests.
