# Supplementary material for: A pharmacogenetics study of platinum-based chemotherapy in lung cancer: ABCG2 polymorphism and its genetic interaction with SLC31A1 are associated with response and survival
Source: J Cancer. 2021 Jan 1;12(5):1270–83. doi: 10.7150/jca.51621 (PMC7847637; doi:10.7150/jca.51621)
Supplement: Supplementary file 1 — Supplementary figures and tables. [file jcav12p1270s1.pdf]

A pharmacogenetics study of platinum-based chemotherapy in lung cancer: *ABCG2* polymorphism and its genetic interaction with *SLC31A1* are associated with response and survival

Liyan Wang, Chang Sun, Xiangnan Li, Chenxue Mao, Ji Qian, Jiucun Wang, Junjie Wu, Qiang Li, Chunxue Bai, Baohui Han, Zhiqiang Gao, Jibin Xu, Jiye Yin, Zhaoqian Liu, Daru Lu, Li Jin and Haijian Wang

Supplemental Materials

Supplemental Table 1. *ABCG2* tag and potentially functional SNPs genotyped in the cohort of NSCLC patients

Supplemental Table 2. Association between *ABCG2* SNPs and response

Supplemental Table 3. Stratification analysis of association between *ABCG2* SNPs and response

Supplemental Table 4. Association between *SLC31A1* rs2233914 (G/A) and response

Supplemental Table 5. The ORR related to the joint effects of *SLC31A1* rs2233914 (G/A) and *ABCG2* rs2231142 (A/C) polymorphisms

Supplemental Table 6. Association between *ABCG2* SNPs and survival

Supplemental Table 7. Stratification analysis of association between *ABCG2* polymorphisms and overall survival

Supplemental Table 8. Joint association of *SLC31A1* rs10759637 (A/C) and *ABCG2* rs2231142 (C/A) with overall survival

Supplemental Table 9. Genotypic distribution of *ABCG2* SNPs between NSCLC patients with mild or severe toxicological outcomes

Supplemental Table 10. Association between *ABCG2* SNPs and toxicity outcomes

Supplemental Table 11. Association between objective response rate and overall survival

Supplemental Table 12. Association between objective response rate and progression free survival

Supplemental Figure 1. Schematic structure of *ABCG2* gene and linkage disequilibrium of its candidate SNPs in the NSCLC cohort

Supplemental Figure 2. Kaplan-Meier curve of estimated overall survival for the NSCLC cohort according to *ABCG2* rs2231142 and *SLC31A1* rs10759637 polymorphisms

Supplemental Table 1. *ABCG2* tag and potentially functional SNPs genotyped in the cohort of NSCLC patients

SNP	Chr4 position	Genic location	Allele (Wild/Variant)	Genotyping rate (%)	Minor allele frequency				*P* value for HWE			*P* value [b]	Effect
					Panel A	Panel B	All	Chinese Han [a]	Panel A	Panel B	All		
rs2231164	88094705	intron	A/G	100.0	0.483	0.468	0.472	0.476	0.683	0.877	1.000	0.599	
rs4148157	88099782	intron	G/A	100.0	0.283	0.258	0.264	0.231	0..904	0.472	0.724	0.017	
rs12505410	88109689	intron	A/C	100.0	0.314	0.342	0.335	0.322	0.266	0.913	0.537	0.493	
rs2231146	88118348	intron	A/G	100.0	0.230	0.242	0.239	0.219	0.210	0.632	0.217	0.322	
rs1871744	88118477	intron	A/G	100.0	0.300	0.317	0.313	0.298	0.172	0.389	0.110	0.503	
rs11931123	88128956	intron	A/C	97.9	0.078	0.092	0.089	0.084	0.564	0.850	0.695	0.959	
rs2231142	88131171	exon	C/A	100.0	0.325	0.315	0.317	0.284	0.823	0.893	0.995	0.299	Gln141Lys
rs2231138	88132566	intron	A/G	100.0	0.076	0.100	0.095	0.103	0.994	0.999	1.000	0.573	
rs17731538	88134227	intron	G/A	100.0	0.051	0.057	0.055	0.065	0.762	0.902	0.771	0.654	
rs2231137	88139962	exon	G/A	72.6	0.340	0.395	0.381	0.322	0.973	0.519	0.672	0.077	Val12Met
rs2725252	88140758	intron	C/A	100.0	0.407	0.364	0.374	0.413	0.913	0.818	0.944	0.319	
rs3109823	88143450	intron	A/G	100.0	0.207	0.226	0.222	0.202	0.286	0.990	0.695	0.666	
rs6857600	88144923	intron	G/A	100.0	0.120	0.134	0.130	0.142	0.996	0.350	0.445	0.830	

[a] Minor allele frequency for the Chinese Han population (n = 208) in the 1000genome database.
[b] Pearson χ^2 tests for overall distributions for SNPs genotypes between the cohort of NSCLC patients in this study and Chinese Han population (n = 208) in the 1000genome database.

Supplemental Table 2. Association between *ABCG2* SNPs and response

SNP	Genotype	Panel A		Panel B		All	
		Response (CR+PR/SD+PD)	*P* value [a]	Response (CR+PR/SD+PD)	*P* value [a]	Response (CR+PR/SD+PD)	*P* value [a]
rs2231164	A/A	16/51	0.274	33/173	**0.028**	49/224	**0.035**
	A/G	16/92		60/316		76/408	
	G/G	12/47		40/119		52/166	
rs4148157	G/G	23/100	0.433	63/335	**0.036**	86/435	**0.043**
	G/A	16/75		57/245		73/320	
	A/A	5/15		13/28		18/43	
rs12505410	A/A	21/96	0.561	69/258	0.131	90/354	0.282
	A/C	15/71		51/272		66/343	
	C/C	8/23		13/78		21/101	
rs2231146	A/A	25/121	0.384	87/348	0.178	112/469	0.541
	A/G	13/55		40/215		53/270	
	G/G	6/14		6/45		12/59	
rs1871744	A/A	25/98	**0.023**	77/279	**0.042**	102/377	**0.022**
	A/G	9/72		44/254		53/326	
	G/G	10/20		12/75		22/95	
rs11931123	A/A	40/159	0.446	111/484	0.485	151/643	0.253
	A/C	4/27		17/102		21/129	
	C/C	0/3		1/7		1/10	
rs2231142	C/C	21/89	0.618	55/292	**0.002** [b]	76/381	**0.005** [b]
	C/A	17/80		55/270		72/350	
	A/A	6/21		23/46		29/67	
rs2231138	A/A	37/162	0.844	106/495	0.846	143/657	0.777
	A/G	7/27		26/107		33/134	
	G/G	0/1		1/6		1/7	
rs17731538	G/G	42/170	0.463	119/538	0.851	161/708	0.535

	G/A	2/18		14/67		16/85	
	A/A	0/2		0/3		0/5	
rs2231137	G/G	15/66	0.385	39/147	0.250	54/213	0.718
	G/A	16/64		49/223		65/287	
	A/A	7/15		9/65		16/80	
rs2725252	C/C	17/63	0.609	63/239	0.191	80/302	0.191
	C/A	22/95		51/282		73/377	
	A/A	5/32		19/87		24/119	
rs3109823	A/A	28/125	0.958	83/364	0.810	111/489	0.892
	A/G	13/52		44/210		57/262	
	G/G	3/13		6/34		9/47	
rs6857600	G/G	36/144	0.542	106/458	0.474	142/602	0.382
	G/A	8/43		23/134		31/177	
	A/A	0/3		4/16		4/19	

[a] *P* values of Pearson χ^2 tests.
[b] Statistical significance remained after multiple tests adjustment taking into account linkage disequilibrium between polymorphisms.

Supplemental Table 3. Stratification analysis of association between *ABCG2* SNPs and response

SNP (Wild/Variant, W/V)	Stratification subgroup		Genotype (WW−WV−VV)		Genetic model [a]	OR (95% CI) [b]	*P* value [b]
			CR+PR	SD+PD			
rs1871744 (A/G)	Gender	Male	79–32–20	262–228–68	Under-DOM	**0.42 (0.27–0.67)**	**2.37×10^{-4}**
		Female	23–21–2	115–98–27	REC	0.48 (0.11–2.16)	0.338
	Age	≤58	41–32–10	195–166–58	DOM	0.86 (0.53–1.42)	0.557
		>58	61–21–12	182–160–37	Under-DOM	**0.39 (0.23–0.68)**	**0.001**
	ECOG PS	0-1	96–45–21	339–294–83	Under-DOM	**0.56 (0.38–0.83)**	**0.003**
		2	6–7–1	32–29–9	REC	0.13 (0.01–3.21)	0.210
	Smoking status	Nonsmoker	36–27–6	165–139–38	REC	0.88 (0.35–2.22)	0.782
		Smoker	66–26–16	211–187–57	DOM	**0.41 (0.25–0.68)**	**0.001**
	TNM stage	ⅢA	17–9–1	26–23–3	DOM	0.48 (0.16–1.40)	0.179
		ⅢB	31–18–7	114–93–21	Under-DOM	0.63 (0.32–1.22)	0.172
		Ⅳ	54–26–14	234–208–71	Under-DOM	**0.59 (0.36–0.97)**	**0.038**
	Histological type	AC	39–30–9	257–215–62	REC	1.21 (0.56–2.60)	0.628
		SCC	38–18–6	62–72–21	DOM	**0.40 (0.21–0.75)**	**0.005**
	Therapy regimens	Pt-navelbine	40–24–9	117–89–23	Under-DOM	0.73 (0.41–1.31)	0.292
		Pt-gemcitabine	22–9–6	83–79–32	DOM	0.54 (0.25–1.16)	0.114
		Pt-paclitaxe	27–16–6	125–112–24	Under-DOM	0.66 (0.34–1.31)	0.237
		Pt-docetaxel	6–4–1	35–30–9	Under-DOM	0.35 (0.06–1.94)	0.227
rs2231142 (C/A)	Gender	Male	59–49–23	265–245–48	REC	**2.19 (1.23–3.92)**	**0.008**
		Female	17–23–6	116–105–19	DOM	1.63 (0.82–3.22)	0.164
	Age	≤58	35–38–10	210–181–28	REC	1.95 (0.87–4.37)	0.106
		>58	41–34–19	171–169–39	REC	**2.49 (1.30–4.78)**	**0.006**
	ECOG PS	0-1	70–65–27	340–317–59	REC	**2.05 (1.28–3.60)**	**0.004**
		2	5–7–2	34–30–6	DOM	2.20 (0.46–10.45)	0.321
	Smoking status	Nonsmoker	26–31–12	163–154–25	REC	**2.35 (1.05–5.23)**	**0.037**
		Smoker	50–41–17	218–196–41	REC	1.84 (0.96–3.53)	0.065

TNM stage	ⅢA	14–9–4	22–24–6	Under-DOM	**0.54 (0.18–1.60)**	**0.267**
	ⅢB	22–25–9	110–91–27	REC	**2.96 (1.51–5.82)**	**0.002**
	Ⅳ	40–38–16	248–231–34	DOM	1.48 (0.79–2.76)	0.220
Histological type	AC	34–35–9	251–237–46	REC	1.35 (0.62–2.94)	0.449
	SCC	27–21–14	81–63–11	REC	**3.56 (1.46–8.66)**	**0.005**
Therapy regimens	Pt-navelbine	31–34–8	108–96–25	DOM	1.39 (0.79–2.43)	0.251
	Pt-gemcitabine	16–15–6	98–87–9	REC	**3.65 (1.12–11.83)**	**0.031**
	Pt-paclitaxe	20–16–13	120–117–24	REC	**3.47 (1.50–8.02)**	**0.004**
	Pt-docetaxel	8–3–0	36–33–5	DOM	**0.12 (0.02–0.93)**	**0.043**

[a] For each SNP, three different genetic models (dominant, recessive and under-dominant) were analyzed, and the model with lowest *P* values was considered the best-fitting model.

[b] Odds ratios (OR) and their 95% confidence intervals (CIs) and *P* values were calculated with unconditional logistic regression analysis, with adjustment of gender, age, smoking status, ECOG performance status, TNM status, histological types, and treatment regimen.

Supplemental Table 4. Association between *SLC31A1* rs2233914 (G/A) and response

Genotype [a]	Response (CR+PR/SD+PD)	*P* value [b]	OR(95%CI) [c]	*P* value [c]
G/G	95/352	0.052	1.00 (ref)	
G/A	66/341		0.73 (0.51–1.05)	0.089
A/A	16/105		**0.51 (0.28–0.92)**	**0.026**
G/A+A/A *vs* G/G	82/446	0.021	**0.67 (0.48–0.95)**	**0.024**

[a] *SLC31A1* rs2233914 (G/A) for the study subjects had been genotyped in our previous report (Ref 9).
[b] *P* values of Pearson χ^2 tests.
[c] Odds ratios (OR) and their 95% confidence intervals (CIs) and *P* values were calculated with unconditional logistic regression analysis, with adjustment of gender, age, smoking status, ECOG performance status, TNM status, histological types, and treatment regimen.

Supplemental Table 5. The ORR related to the joint effects of *SLC31A1* rs2233914 (G/A) and *ABCG2* rs2231142 (A/C) polymorphisms

	SLC31A1 (rs2233914) [a]					
	G/G			A/G+A/A		
	Response (CR+PR/SD+PD)	OR(95%CI) [b]	*P* value [a]	Response (CR+PR/SD+PD)	OR(95%CI) [b]	*P* value [b]
ABCG2 (rs2231142)						
A/A	12/31	1.00 (ref)		17/36	1.34 (0.52–3.43)	0.547
C/C+A/C	83/321	0.74 (0.35–1.57)	0.429	65/410	0.44 (0.20–0.95)	**0.036**

[a] *SLC31A1* rs2233914 (G/A) for the study subjects had been genotyped in our previous report (Ref 9).
[a] Odds ratios (OR) and their 95% confidence intervals (CIs) and *P* values were calculated with unconditional logistic regression analysis, with adjustment of gender, age, smoking status, ECOG performance status, TNM status, histological types, and treatment regimen.

Supplemental Table 6. Association between *ABCG2* SNPs and survival

		Overall survival					Progression-free survival				
SNP	Genotype	n/N [a]	MST (m) [b]	Log-rank *P*	HR (95% CI) [c]	*P* [c]	n/N [d]	MST (m) [b]	Log-rank *P*	HR (95% CI) [c]	*P* [c]
**Panel A**											
rs2231164	A/A	47/65	22.4	**0.025**	1.00 (ref)		30/47	9.1	0.951	1.00 (ref)	
	A/G	79/104	19.3		1.36 (0.92–2.00)	0.123	47/77	9.8		0.88 (0.52–1.47)	0.614
	G/G	47/59	15.8		**1.86 (1.18–2.94)**	**0.015**	22/41	10.8		0.86 (0.53–2.15)	0.613
rs4148157	G/G	84/118	21.8	**0.018**	1.00 (ref)		51/81	9.1	0.991	1.00 (ref)	
	G/A	72/90	15.6		1.35 (0.95–1.92)	0.093	38/67	9.8		0.92 (0.59–1.43)	0.705
	A/A	17/0	15.1		1.67 (0.95–2.93)	0.075	10/17	7.1		1.08 (0.52–2.25)	0.844
	G/A+A/A *vs* G/G	89/110	15.4	**0.006**	**1.40 (1.01–1.96)**	**0.045**	48/84	9.8	0.962	0.95 (0.62–1.44)	0.793
rs12505410	A/A	92/114	18.7	0.083	1.00 (ref)		45/88	10.2	0.137	1.00 (ref)	
	A/C	60/85	21.4		0.73 (0.51–1.04)	0.079	42/57	6.9		1.43 (0.89–2.28)	0.138
	C/C	21/29	21.5		0.82 (0.49–1.36)	0.437	12/20	12.0		1.08 (0.55–2.13)	0.816
rs2231146	A/A	112/142	19.3	0.130	1.00 (ref)		54/103	10.2	0.153	1.00 (ref)	
	A/G	49/68	21.0		0.81 (0.57–1.16)	0.252	38/52	7.1		1.34 (0.85–2.13)	0.211
	G/G	12/18	21.7		0.67 (0.36–1.25)	0.202	7/10	10.1		1.22 (0.52–2.89)	0.644
rs1871744	A/A	95/121	19.3	0.159	1.00 (ref)		46/91	10.2	**0.025**	1.00 (ref)	
	A/G	61/80	20.9		0.82 (0.58–1.16)	0.263	43/57	6.9		1.54 (0.97–2.44)	0.070
	G/G	17/27	21.7		0.68 (0.40–1.16)	0.153	10/17	11.1		0.88 (0.42–1.82)	0.724
rs11931123	A/A	148/195	20.7	0.577	1.00 (ref)		85/143	9.8	0.778	1.00 (ref)	
	A/C	24/30	19.9		0.94 (0.60–1.49)	0.806	12/19	6.6		1.15 (0.59–2.25)	0.677
	C/C	0/2	NA		NA	NA	1/2	9.3		0.46 (0.06–3.57)	0.457
rs2231142	C/C	76/106	21.7	**0.025**	1.00 (ref)		47/74	9.1	0.791	1.00 (ref)	

	C/A	73/95	15.4		**1.49 (1.05–2.10)**	**0024**	38/70	10.2		0.92 (0.58–1.43)	0.699
	A/A	24/27	21.2		1.46 (0.88–2.42)	0.142	14/21	7.1		1.28 (0.67–2.43)	0.453
	C/A+A/A *vs* C/C	97/122	16.6	**0.007**	**1.48 (1.07–2.06)**	**0.019**	52/91	9.8	0.885	0.99 (0.66–1.50)	0.965
rs2231138	A/A	150/194	19.8	0.158	1.00 (ref)		84/139	9.3	0.413	1.00 (ref)	
	A/G	23/33	23.0		0.80 (0.5–1.27)	0.334	15/26	12.0		0.71 (0.39–1.27)	0.245
	G/G	0/1	NA		NA	NA	0/0	NA		NA	NA
rs17731538	G/G	157/208	20.4	0.643	1.00 (ref)		92/153	9.5	0.549	1.00 (ref)	
	G/A	16/19	19.9		1.07 (0.61–1.85)	0.923	7/11	9.3		1.05 (0.43–2.55)	0.916
	A/A	0/1	NA		NA	NA	0/1			NA	NA
rs2231137	G/G	61/75	19.9	0.110	1.00 (ref)		31/61	10.8	0.322	1.00 (ref)	
	G/A	58/80	21.4		0.98 (0.66–1.45)	0.913	38/59	7.5		1.02 (0.59–1.78)	0.942
	A/A	13/21	23.0		0.68 (0.35–1.32)	0.252	7/14	13.1		0.63 (0.26–1.53)	0.305
rs2725252	C/C	59/78	21.5	0.715	1.00 (ref)		38/57	7.7	0.666	1.00 (ref)	
	C/A	86/116	19.3		1.12 (0.79–1.60)	0.535	49/86	10.2		1.08 (0.68–1.72)	0.748
	A/A	28/34	20.9		1.04 (0.65–1.69)	0.863	12/22	9.3		0.87 (0.42–1.81)	0.715
rs3109823	A/A	119/149	19.6	0.179	1.00 (ref)		58/107	10.2	0.316	1.00 (ref)	
	A/G	45/65	21.4		0.83 (0.57–1.21)	0.332	36/51	7.2		1.25 (0.80–1.96)	0.320
	G/G	9/14	21.7		0.73 (0.36–1.47)	0.375	5/7	7.7		1.06 (0.41–2.76)	0.905
rs6857600	G/G	138/176	19.6	0.095	1.00 (ref)		75/126	9.5	0.826	1.00 (ref)	
	G/A	35/50	23.0		0.69 (0.46–1.02)	0.063	23/37	9.1		0.93 (0.57–1.52)	0.771
	A/A	0/2	NA		NA	NA	1/2	9.3		0.43 (0.06–3.37)	0.423
**Panel B**											
rs2231164	A/A	152/205	19.1	0.383	1.00 (ref)		126/200	7.7	0.660	1.00 (ref)	
	A/G	276/379	18.8		1.01 (0.83–1.24)	0.915	237/371	8.8		0.98 (0.79–1.22)	0.883
	G/G	127/160	18.2		1.15 (0.91–1.47)	0.242	96/160	10.2		0.86 (0.66–1.13)	0.286

rs4148157	G/G	287/402	19.0	0.120	1.00 (ref)		238/392	9.1	0.634	1.00 (ref)	
	G/A	236/300	18.9		**1.20 (1.00–1.43)**	**0.047**	196/297	8.2		1.09 (0.90–1.32)	0.380
	A/A	32/42	20.2		1.14 (0.78–1.66)	0.507	25/42	9.1		1.01 (0.65–1.56)	0.968
	G/A+A/A *vs* G/G	268/342	18.9	**0.040**	**1.19 (1.01–1.41)**	**0.048**	221/339	8.8	0.373	1.08 (0.90–1.30)	0.415
rs12505410	A/A	245/327	17.0	0.350	1.00 (ref)		210/323	8.0	0.581	1.00 (ref)	
	A/C	241/327	19.7		0.85 (0.71–1.03)	0.093	195/320	10.1		0.86 (0.70–1.05)	0.143
	C/C	69/90	22.2		0.78 (0.59–1.03)	0.075	54/88	8.0		0.75 (0.55–1.03)	0.074
rs2231146	A/A	320/436	17.9	0.725	1.00 (ref)		273/431	9.1	0.942	1.00 (ref)	
	A/G	198/258	19.7		0.97 (0.80–1.16)	0.718	153/251	9.1		0.94 (0.77–1.16)	0.569
	G/G	37/50	22.2		0.87 (0.62–1.24)	0.446	33/49	7.7		0.89 (0.61–1.29)	0.533
rs1871744	A/A	257/359	18.3	0.750	1.00 (ref)		228/355	9.1	0.884	1.00 (ref)	
	A/G	235/299	19.1		1.06 (0.89–1.27)	0.520	178/291	9.1		0.96 (0.78–1.17)	0.656
	G/G	63/86	22.2		0.99 (0.75–1.33)	0.969	53/85	8.0		0.86 (0.63–1.17)	0.338
rs11931123	A/A	441/599	19.1	0.063	1.00 (ref)		369/588	9.0	0.466	1.00 (ref)	
	A/C	96/117	17.8		**1.39 (1.10–1.74)**	**0.005**	68/116	9.2		0.93 (0.71–1.21)	0.568
	C/C	5/8	15.9		0.86 (0.35–2.11)	0.734	6/8	5.3		1.39 (0.61–3.19)	0.435
rs2231142	C/C	248/327	19.0	0.326	1.00 (ref)		206/338	8.8	0.807	1.00 (ref)	
	C/A	248/329	18.9		1.06 (0.88–1.27)	0.541	208/322	9.2		1.07 (0.88–1.30)	0.496
	A/A	60/76	16.5		1.21 (0.90–1.63)	0.207	45/71	8.2		1.06 (0.76–1.49)	0.735
	C/A+A/A *vs* C/C	308/405	18.9	0.336	1.08 (0.91–1.29)	0.363	253/393	9.1	0.515	1.07 (0.89–1.29)	0.488
rs2231138	A/A	453/601	18.6	0.412	1.00 (ref)		371/590	8.8	0.925	1.00 (ref)	
	A/G	99/137	19.3		0.85 (0.68–1.07)	0.163	85/135	9.3		1.01 (0.79–1.29)	0.934
	G/G	3/6	43.8		0.55 (0.18–1.75)	0.314	3/6	7.1		0.80 (0.25–2.51)	0.698
rs17731538	G/G	491/661	19.0	0.513	1.00 (ref)		413/649	9.0	0.468	1.00 (ref)	
	G/A	62/80	17.8		1.20 (0.91–1.57)	0.201	44/79	9.6		0.91 (0.66–1.26)	0.580

	A/A	2/3	24.3		1.90 (0.46–7.86)	0.374	2/3	13.7		1.13 (0.27–4.64)	0.868
rs2231137	G/G	140/185	17.9	0.858	1.00 (ref)		122/184	7.4	0.917	1.00 (ref)	
	G/A	199/269	19.1		0.95 (0.76–1.19)	0.641	171/267	8.8		0.97 (0.77–1.24)	0.822
	A/A	57/72	20.4		0.88 (0.64–1.21)	0.439	45/71	8.0		0.84 (0.59–1.19)	0.329
rs2725252	C/C	234/304	19.1	0.831	1.00 (ref)		191/298	9.0	0.668	1.00 (ref)	
	C/A	248/335	18.7		1.01 (0.84–1.22)	0.885	210/331	8.8		1.03 (0.84–1.27)	0.750
	A/A	73/105	19.1		0.99 (0.75–1.29)	0.925	58/102	9.7		0.93 (0.69–1.25)	0.639
rs3109823	A/A	329/448	19.1	0.937	1.00 (ref)		275/442	9.0	0.752	1.00 (ref)	
	A/G	195/257	18.8		1.02 (0.85–1.22)	0.829	159/251	9.2		1.04 (0.85–1.28)	0.677
	G/G	31/39	18.8		1.01 (0.68–1.49)	0.974	25/38	7.4		0.82 (0.53–1.26)	0.361
rs6857600	G/G	426/566	18.9	0.909	1.00 (ref)		348/554	9.1	0.600	1.00 (ref)	
	G/A	116/158	19.3		0.97 (0.78–1.19)	0.739	101/157	8.0		1.01 (0.81–1.27)	0.929
	A/A	13/20	17.8		0.87 (0.50–1.53)	0.631	10/20	11.9		0.75 (0.40–1.43)	0.382
**All**											
rs2231164	A/A	199/270	20.7	**0.039**	1.00 (ref)		156/247	7.7	0.636	1.00 (ref)	
	A/G	355/483	19.0		1.08 (0.91–1.29)	0.377	284/448	9.2		0.99 (0.82–1.21)	0.954
	G/G	174/219	18.0		**1.26 (1.02–1.55)**	**0.031**	118/201	10.2		0.88 (0.69–1.12)	0.282
rs4148157	G/G	371/520	20.4	**0.008** [e]	1.00 (ref)		289/473	9.1	0.672	1.00 (ref)	
	G/A	308/390	17.9		**1.21 (1.04–1.42)**	**0.014**	234/364	9.1		1.09 (0.92–1.30)	0.336
	A/A	49/62	17.0		1.25 (0.92–1.70)	0.163	35/59	9.1		1.03 (0.71–1.49)	0.883
	G/A+A/A *vs* G/G	357/452	17.9	**0.002**	**1.22 (1.05–1.42)**	**0.010**	269/423	9.1	0.400	1.08 (0.91–1.28)	0.362
rs12505410	A/A	337/441	17.1	0.064	1.00 (ref)		255/411	9.1	0.794	1.00 (ref)	
	A/C	301/412	20.7		**0.84 (0.72–0.99)**	**0.039**	237/377	9.3		0.96 (0.80–1.15)	0.638
	C/C	90/119	21.6		**0.77 (0.61–0.96)**	**0.034**	66/108	9.1		0.84 (0.63–1.11)	0.209
rs2231146	A/A	432/578	18.2	0.218	1.00 (ref)		327/534	9.2	0.707	1.00 (ref)	

	A/G	247/326	20.7		0.94 (0.80–1.11)	0.456	191/303	8.8		1.02 (0.85–1.23)	0.833
	G/G	49/68	21.8		0.79 (0.58–1.06)	0.118	40/59	7.7		0.98 (0.70–1.37)	0.882
rs1871744	A/A	352/480	18.9	0.532	1.00 (ref)		274/446	9.2	0.593	1.00 (ref)	
	A/G	296/379	19.7		1.02 (0.87–1.19)	0.848	221/348	8.3		1.04 (0.87–1.25)	0.651
	G/G	80/113	21.7		0.89 (0.70–1.15)	0.379	63/102	9.4		0.91 (0.69–1.21)	0.525
rs11931123	A/A	589/794	19.5	0.120	1.00 (ref)		454/731	9.1	0.694	1.00 (ref)	
	A/C	120/147	17.9		**1.28 (1.05–1.57)**	**0.016**	80/135	9.1		0.96 (0.75–1.23)	0.746
	C/C	5/10	24.3		0.79 (0.32–1.92)	0.598	7/10	9.3		1.09 (0.51–2.33)	0.828
rs2231142	C/C	327/452	20.4	**0.043**	1.00 (ref)		253/412	9.1	0.745	1.00 (ref)	
	C/A	321/422	17.9		1.14 (0.97–1.33)	0.110	246/392	9.5		1.06 (0.89–1.26)	0.539
	A/A	80/98	19.6		1.27 (0.99–1.64)	0.064	59/92	7.2		1.11 (0.83–1.49)	0.487
	C/A+A/A *vs* C/C	401/520	18.0	**0.028**	1.16 (1.00–1.35)	0.051	305/484	9.2	0.488	1.07 (0.90–1.26)	0.454
rs2231138	A/A	603/795	19.0	0.111	1.00 (ref)		455/729	9.1	0.822	1.00 (ref)	
	A/G	122/170	20.4		0.83 (0.68–1.01)	0.067	100/161	9.3		0.96 (0.77–1.19)	0.685
	G/G	3/7	44.2		0.44 (0.14–1.37)	0.157	3/6	7.1		0.76 (0.24–2.39)	0.639
rs17731538	G/G	648/869	19.3	0.606	1.00 (ref)		505/802	9.1	0.487	1.00 (ref)	
	G/A	78/99	17.9		1.20 (0.94–1.53)	0.136	51/90	9.3		0.94 (0.70–1.26)	0.664
	A/A	2/4	24.3		1.23 (0.30–5.00)	0.778	2/4	13.7		0.59 (0.15–2.43)	0.467
rs2231137	G/G	201/260	18.3	0.307	1.00 (ref)		153/245	7.8	0.693	1.00 (ref)	
	G/A	257/349	19.7		0.94 (0.78–1.13)	0.504	209/326	8.7		1.01 (0.82–1.26)	0.896
	A/A	70/93	21.7		0.81 (0.61–1.07)	0.137	52/85	9.4		0.83 (0.60–1.14)	0.254
rs2725252	C/C	293/382	20.2	0.964	1.00 (ref)		229/355	8.7	0.471	1.00 (ref)	
	C/A	334/451	18.9		1.03 (0.88–1.21)	0.709	259/417	9.2		1.03 (0.86–1.24)	0.725
	A/A	101/139	19.8		1.01 (0.80–1.27)	0.940	70/124	9.7		0.88 (0.67–1.16)	0.357
rs3109823	A/A	448/597	19.3	0.682	1.00 (ref)		333/549	9.1	0.437	1.00 (ref)	

	A/G	240/322	19.3		0.99 (0.85–1.17)	0.945	195/302	9.2		1.10 (0.92–1.32)	0.319
	G/G	40/53	19.0		0.88 (0.63–1.23)	0.449	30/45	7.4		0.88 (0.60–1.30)	0.525
rs6857600	G/G	564/742	19.1	0.340	1.00 (ref)		423/680	9.1	0.522	1.00 (ref)	
	G/A	151/208	19.8		0.89 (0.74–1.06)	0.197	124/194	8.0		1.00 (0.82–1.23)	0.969
	A/A	13/22	17.8		0.85 (0.49–1.47)	0.551	11/22	11.9		0.72 (0.39–1.32)	0.285

[a] Numbers indicate the death event for NSCLC patients during the following-up time among all individuals in the same genotype group.
[b] MST: median survival time.
[c] Hazard ratios (HR) and their 95% confidence intervals (CIs) and *P* values were calculated with by multivariate Cox proportional hazards regression with adjustment for covariates.
[d] Numbers indicate patients who suffered of disease progression (including death) during the following-up time among all individuals in the same genotype group.
[e] Statistical significance remained after Bonferroni multiple tests.

Supplemental Table 7. Stratification analysis of association between *ABCG2* polymorphisms and overall survival

SNP (Wild/Variant, W/V)	Stratification subgroup		Overall survival								
			n/N [a]			MST (m) [b]			Log-rank	HR (95%CI) [d]	*P* [d]
			WW	WV	VV	WW	WV	VV	*P* [c]		
rs4148157 (G/A)	Gender	Male	266/364	226/278	36/47	19.4	17.4	16.5	0.133	1.19 (1.00–1.42)	0.057
		Female	105/156	82/112	13/15	25.2	20.3	8.4	**0.012** [e]	**1.37 (1.01–1.84)**	**0.041**
	Age	≤58	189/283	153/199	17/23	22.5	19.6	21.4	**0.017** [e]	**1.26 (1.01–1.56)**	**0.037**
		>58	182/237	155/191	32/39	18.7	15.7	16.5	0.465	1.15 (0.93–1.42)	0.200
	Smoking status	Smoker	222/302	182/222	29/39	19.5	16.5	21.5	0.077	**1.23 (1.01–1.49)**	**0.038**
		Nonsmoker	148/217	124/166	19/22	22.7	20.0	17.0	**0.021**	1.18 (0.93–1.51)	0.178
	ECOG PS	0-1	328/468	285/356	43/55	21.0	17.7	16.5	**0.002** [e]	**1.26 (1.08–1.48)**	**0.004**
		2	35/43	21/31	5/6	12.4	19.1	11.8	0.608	0.60 (0.33–1.10)	0.101
	TNM stage	ⅢA	29/41	22/30	5/5	28.8	19.3	23.6	0.546	1.85 (0.98–3.51)	0.060
		ⅢB	107/146	87/108	21/29	21.6	16.0	15.3	**0.004** [e]	**1.50 (1.14–1.99)**	**0.004**
		Ⅳ	234/332	196/248	23/28	18.9	19.0	21.9	0.388	1.11 (0.91–1.34)	0.303
	Histologic type	AC	236/333	186/240	32/39	21.9	18.2	14.4	**0.006** [e]	**1.30 (1.08–1.57)**	**0.006**
		SCC	82/116	70/85	9/12	15.7	17.0	20.2	0.992	0.93 (0.66–1.30)	0.654
	Therapy regimens	Pt-navelbine	116/162	95/122	18/22	21.9	18.4	21.9	0.521	1.07 (0.82–1.40)	0.617
		Pt-gemcitabine	89/130	76/96	9/10	22.5	17.6	6.0	**3.20×10^{-5}** [e]	**1.58 (1.15–2.18)**	**0.005**
		Pt-paclitaxe	115/155	97/122	16/23	18.3	17.7	21.5	0.279	0.99 (0.75–1.30)	0.928
		Pt-docetaxel	37/51	24/29	3/3	17.7	22.0	16.4	0.537	1.01 (0.61–1.70)	0.958
rs2231142 (C/A)	Gender	Male	236/322	235/296	57/71	19.5	17.2	20.2	0.270	1.15 (0.96–1.37)	0.128
		Female	91/130	86/126	23/27	24.9	22.8	17.0	**0.017** [e]	1.28 (0.95–1.72)	0.104
	Age	≤58	169/244	159/220	31/41	22.5	19.1	21.9	0.137	1.13 (0.92–1.40)	0.253
		>58	158/208	162/202	49/57	18.2	17.0	16.4	0.391	1.17 (0.95–1.45)	0.145
	Smoking status	Smoker	197/266	189/238	47/59	19.5	17.0	21.5	0.245	1.19 (0.97–1.45)	0.092
		Nonsmoker	129/185	130/182	32/38	22.5	20.2	19.6	0.065	1.13 (0.88–1.43)	0.336
	ECOG PS	0-1	289/407	294/384	73/88	21.0	17.9	17.0	**0.014** [e]	**1.19 (1.02–1.40)**	**0.027**
		2	31/37	24/35	6/8	12.4	19.1	21.4	0.674	0.63 (0.32–1.22)	0.168

TNM stage	ⅢA	23/34	24/33	9/9	28.8	18.9	25.5	0.588	1.80 (0.93–3.48)	0.081
	ⅢB	98/131	89/114	28/38	20.9	17.0	15.3	**0.017** [e]	**1.46 (1.10–1.94)**	**0.009**
	Ⅳ	205/286	205/271	43/51	19.3	18.0	21.7	0.522	1.06 (0.87–1.28)	0.574
Histologic type	AC	204/283	203/272	47/57	22.3	18.2	16.4	**0.032**	**1.24 (1.03–1.50)**	**0.025**
	SCC	77/107	64/82	20/24	14.6	17.0	23.6	0.992	0.89 (0.63–1.25)	0.488
Therapy regimens	Pt-navelbine	98/141	104/132	27/33	22.2	17.1	23.5	0.780	1.12 (0.85–1.47)	0.415
	Pt-gemcitabine	79/115	80/105	15/16	22.5	19.3	8.4	**4.85×10^{-4}** [e]	**1.46 (1.06–2.02)**	**0.020**
	Pt-paclitaxe	106/135	94/128	28/37	18.3	17.7	21.3	0.875	0.88 (0.67–1.15)	0.339
	Pt-docetaxel	31/43	28/35	5/5	20.4	17.4	17.0	0.628	1.25 (0.75–2.10)	0.401

[a] Numbers indicate the death event for NSCLC patients during the following-up time among all individuals in the same genotype group.
[b] MST: median survival time.
[c] Log-rank tests for association between survival and overall genotypes.
[d] Hazard ratios (HR) and their 95% confidence intervals (CIs) and *P* values were calculated with by multivariate Cox proportional hazards regression with adjustment for covariates. Dominant models were used to estimate the HRs for the two SNPs.
[e] Statistical significance remained after Bonferroni multiple tests.

Supplemental Table 8. Joint association of *SLC31A1* rs10759637 (A/C) and *ABCG2* rs2231142 (C/A) with overall survival

Stratification subgroup		Genotype (*SLC31A1*—*ABCG2*) [a]	n/N [b]	MST (m) [c]	Log-rank *P*	HR (95% CI) [d]	*P* value [d]
All		[A/A+C/C] — [C/C]	168/237	20.2	**5.20×10^{-5}** [f]	1.00 (ref)	
		[A/C] — [C/C]	159/215	20.7		1.01 (0.81–1.27)	0.917
		[A/A+C/C] — [C/A+A/A]	204/285	19.9		0.98 (0.79–1.20)	0.818
		[A/C] — [C/A+A/A]	197/235	15.7		**1.47 (1.19–1.81)** [e]	**3.84×10^{-4}**
Gender	Male	[A/A+C/C] — [C/C]	129/174	19.5	**0.001** [f]	1.00 (ref)	
		[A/C] — [C/C]	107/148	19.8		0.97 (0.75–1.27)	0.839
		[A/A+C/C] — [C/A+A/A]	152/206	19.5		0.95 (0.74–1.21)	0.655
		[A/C] — [C/A+A/A]	140/161	14.9		**1.46 (1.14–1.87)**	**0.003**
	Female	[A/A+C/C] — [C/C]	39/63	26.2	**0.049**	1.00 (ref)	
		[A/C] — [C/C]	52/67	23.3		1.12 (0.72–1.74)	0.616
		[A/A+C/C] — [C/A+A/A]	52/79	24.4		1.17 (0.76–1.81)	0.482
		[A/C] — [C/A+A/A]	57/74	18.9		**1.57 (1.02–2.42)**	**0.039**
Age	≤58	[A/A+C/C] — [C/C]	90/129	22.3	0.063	1.00 (ref)	
		[A/C] — [C/C]	79/115	23.3		0.91 (0.66–1.24)	0.532
		[A/A+C/C] — [C/A+A/A]	107/156	21.2		0.93 (0.70–1.24)	0.626
		[A/C] — [C/A+A/A]	83/105	17.9		1.35 (0.99–1.84)	0.057
	>58	[A/A+C/C] — [C/C]	78/108	18.8	**0.003** [f]	1.00 (ref)	
		[A/C] — [C/C]	80/100	16.4		1.08 (0.78–1.50)	0.632
		[A/A+C/C] — [C/A+A/A]	97/129	19.3		0.97 (0.71–1.32)	0.837
		[A/C] — [C/A+A/A]	114/130	14.7		**1.56 (1.16–2.11)**	**0.003**
ECOG PS	0-1	[A/A+C/C] — [C/C]	153/218	20.4	**1.50×10^{-5}** [f]	1.00 (ref)	
		[A/C] — [C/C]	136/189	21.9		1.00 (0.79–1.27)	0.991
		[A/A+C/C] — [C/A+A/A]	187/258	20.2		0.99 (0.80–1.23)	0.945
		[A/C] — [C/A+A/A]	180/214	15.3		**1.50 (1.20–1.87)**	**3.02×10^{-4}**
	2	[A/A+C/C] — [C/C]	11/14	17.8	0.776	1.00 (ref)	

		[A/C] — [C/C]	20/23	10.8		0.87 (0.34–2.18)	0.763
		[A/A+C/C] — [C/A+A/A]	16/26	19.1		0.48 (0.19–1.26)	0.137
		[A/C] — [C/A+A/A]	14/17	19.4		0.76 (0.28–2.06)	0.586
Smoking status	Nonsmoker	[A/A+C/C] — [C/C]	63/97	22.3	0.090	1.00 (ref)	
		[A/C] — [C/C]	66/88	23.8		0.99 (0.69–1.41)	0.952
		[A/A+C/C] — [C/A+A/A]	84/120	21.2		0.97 (0.69–1.37)	0.868
		[A/C] — [C/A+A/A]	78/100	18.9		1.32 (0.94–1.86)	0.115
	Smoker	[A/A+C/C] — [C/C]	105/140	19.8	**0.001** [f]	1.00 (ref)	
		[A/C] — [C/C]	92/126	18.7		1.03 (0.77–1.37)	0.854
		[A/A+C/C] — [C/A+A/A]	119/164	19.2		0.99 (0.75–1.30)	0.914
		[A/C] — [C/A+A/A]	117/133	14.3		**1.57 (1.19–2.08)**	**0.001**
TNM stage	IIIA	[A/A+C/C] — [C/C]	11/16	31.3	0.120	1.00 (ref)	
		[A/C] — [C/C]	12/18	15.7		2.26 (0.79–6.45)	0.127
		[A/A+C/C] — [C/A+A/A]	16/25	27.4		2.51 (0.93–6.81)	0.070
		[A/C] — [C/A+A/A]	17/17	16.5		**3.75 (1.27–11.08)**	**0.017**
	IIIB	[A/A+C/C] — [C/C]	58/78	21.0	**0.033**	1.00 (ref)	
		[A/C] — [C/C]	40/53	20.9		1.05 (0.69–1.61)	0.815
		[A/A+C/C] — [C/A+A/A]	61/86	17.0		1.41 (0.97–2.04)	0.074
		[A/C] — [C/A+A/A]	56/66	15.3		**1.66 (1.13–2.45)**	**0.010**
	IV	[A/A+C/C] — [C/C]	99/143	18.8	**0.007** [f]	1.00 (ref)	
		[A/C] — [C/C]	106/143	20.0		0.92 (0.70–1.22)	0.563
		[A/A+C/C] — [C/A+A/A]	126/172	21.3		0.82 (0.63–1.08)	0.155
		[A/C] — [C/A+A/A]	122/150	16.1		1.30 (0.99–1.70)	0.060
Histological type	AC	[A/A+C/C] — [C/C]	103/147	21.0	**0.005** [f]	1.00 (ref)	
		[A/C] — [C/C]	101/136	23.6		0.91 (0.69–1.21)	0.518
		[A/A+C/C] — [C/A+A/A]	127/176	19.6		1.04 (0.80–1.35)	0.794
		[A/C] — [C/A+A/A]	123/153	16.8		**1.39 (1.06–1.82)**	**0.016**
	SCC	[A/A+C/C] — [C/C]	44/60	18.8	0.071	1.00 (ref)	
		[A/C] — [C/C]	33/47	13.0		1.45 (0.90–2.35)	0.130
		[A/A+C/C] — [C/A+A/A]	41/60	22.0		0.81 (0.52–1.28)	0.375

		[A/C] — [C/A+A/A]	43/46	15.2		1.38 (0.86–2.20)	0.180
Therapy regimens	Pt-navelbine	[A/A+C/C] — [C/C]	50/74	21.8	0.111	1.00 (ref)	
		[A/C] — [C/C]	48/67	23.6		1.01 (0.67–1.52)	0.969
		[A/A+C/C] — [C/A+A/A]	74/100	19.9		0.93 (0.64–1.35)	0.686
		[A/C] — [C/A+A/A]	57/65	16.3		**1.50 (1.02–2.21)**	**0.041**
	Pt-gemcitabine	[A/A+C/C] — [C/C]	34/56	24.7	**0.013**	1.00 (ref)	
		[A/C] — [C/C]	45/59	22.5		1.21 (0.76–1.91)	0.428
		[A/A+C/C] — [C/A+A/A]	46/63	19.1		1.40 (0.88–2.21)	0.154
		[A/C] — [C/A+A/A]	49/58	15.7		**1.99 (1.24–3.22)**	**0.005**
	Pt-paclitaxe	[A/A+C/C] — [C/C]	59/71	16.0	**0.044**	1.00 (ref)	
		[A/C] — [C/C]	47/64	19.8		0.80 (0.53–1.18)	0.259
		[A/A+C/C] — [C/A+A/A]	61/88	21.3		**0.67 (0.46–0.96)**	**0.030**
		[A/C] — [C/A+A/A]	61/77	14.2		0.98 (0.68–1.43)	0.929
	Pt-docetaxel	[A/A+C/C] — [C/C]	18/25	20.9	0.466	1.00 (ref)	
		[A/C] — [C/C]	13/18	17.7		1.70 (0.70–4.15)	0.241
		[A/A+C/C] — [C/A+A/A]	12/18	17.0		1.00 (0.46–2.17)	0.996
		[A/C] — [C/A+A/A]				**2.06 (1.00–4.25)**	**0.050**

[a] *SLC31A1* rs2233914 (G/A) for the study subjects had been genotyped in our previous report (Ref 9).
[b] Numbers indicate the death event for NSCLC patients during the following-up time among all individuals in the same genotype group.
[c] MST: median survival time.
[d] Hazard ratios (HR) and their 95% confidence intervals (CIs) and *P* values were calculated with by multivariate Cox proportional hazards regression with adjustment for covariates.
[e] Test of interaction for the cohort of all patients with *P* value being 0.007.
[f] Statistical significance remained after Bonferroni multiple tests.

Supplemental Table 9. Genotypic distribution of *ABCG2* SNPs between NSCLC patients with mild or severe toxicological outcomes

Toxicological phenotype and grade (G)		SNP genotype (Wild/Variant, WW–WV–VV)												
		rs2231164 (A/G)	rs4148157 (G/A)	rs12505410 (A/C)	rs2231146 (A/G)	rs1871744 (A/G)	rs11931123 (A/C)	rs2231142 (C/A)	rs2231138 (A/G)	rs17731538 (G/A)	rs2231137 (G/A)	rs2725252 (C/A)	rs3109823 (A/G)	rs6857600 (G/A)
Gastrointestinal toxicity	G0+G2	246–438–200	470–357–57	410–364–110	537–286–61	443–341–100	721–135–10	413–383–88	721–155–8	790–90–4	242–316–88	343–411–130	549–288–47	674–190–20
	G3+G4	20–42–18	41–34–5	32–39–9	41–33–6	35–32–13	63–13–1	34–37–9	69–11–0	71–8–1	18–31–4	35–36–9	46–30–4	61–16–3
	P value [a]	0.843	0.934	0.418	0.235	0.339	0.948	0.760	0.444	0.716	0.295	0.577	0.669	0.685
Hematologic toxicity	G0+G2	198–383–156	388–306–43	344–310–83	442–246–49	359–294–84	589–121–9	339–332–66	609–121–7	650–82–5	192–265–66	285–341–111	452–247–38	553–165–19
	G3+G4	71–102–59	130–85–17	98–96–38	134–78–20	115–87–30	199–28–2	112–92–28	185–46–1	215–17–0	67–83–28	94–109–29	145–72–15	183–45–4
	P value [a]	0.101	0.358	0.106	0.573	0.728	0.215	0.209	0.378	0.090	0.490	0.614	0.631	0.446
Anemia	G0+G2	251–461–203	485–374–56	421–380–114	543–309–63	443–364–108	735–149–10	427–397–91	755–153–7	812–99–4	243–324–88	356–425–134	560–305–50	690–203–22
	G3+G4	8–12–9	12–13–4	12–14–3	20–6–3	20–6–3	29–0–0	8–18–3	19–10–0	29–0–0	9–12–3	15–14–0	21–5–3	25–4–0
	P value [a]	0.488	0.180	0.764	0.307	0.079	0.057	0.108	0.045	0.171	0.991	0.066	0.136	0.339
Leukopenia	G0+G2	229–421–181	437–341–53	383–347–101	497–278–56	410–324–97	627–130–10	383–366–82	684–138–9	737–89–5	219–300–79	328–382–121	512–275–44	631–179–21
	G3+G4	44–68–37	85–55–9	67–62–20	89–48–12	74–59–16	127–19–1	70–64–15	120–29–0	139–10–0	46–51–16	56–72–21	93–48–8	115–31–3
	P value [a]	0.511	0.599	0.905	0.830	0.946	0.525	0.970	0.322	0.187	0.613	0.867	0.978	0.908
Neutropenia	G0+G2	225–420–175	431–341–48	382–345–93	494–271–55	402–323–95	655–137–9	377–369–74	677–137–6	724–91–5	218–292–73	315–383–122	508–266–46	615–185–20
	G3+G4	31–51–33	62–43–10	45–48–22	63–42–10	57–42–16	101–11–1	56–41–18	89–25–1	108–7–0	31–44–16	53–49–13	69–40–6	91–22–2
	P value [a]	0.182	0.411	0.045	0.486	0.712	0.131	0.036	0.400	0.203	0.405	0.251	0.878	0.614
Thrombocytopenia	G0+G2	249–463–204	481–377–58	422–384–110	546–309–61	450–361–105	742–143–10	420–406–90	751–158–7	815–96–5	241–330–86	360–426–133	565–302–49	694–200–22
	G3+G4	12–12–10	21–11–2	14–13–7	21–8–5	16–11–7	27–6–1	17–13–4	28–6–0	31–3–0	12–9–5	13–18–3	21–9–4	26–7–1
	P value [a]	0.217	0.559	0.327	0.128	0.251	0.506	0.770	1.000	0.831	0.281	0.584	0.242	1.000
Overall toxicity	G0+G2	181–342–144	353–275–39	316–275–76	410–213–44	333–260–74	532–110–8	311–297–59	546–114–7	590–73–4	176–239–62	255–308–104	416–217–34	499–151–17
	G3+G4	85–132–68	157–108–20	119–122–44	158–103–24	135–111–39	241–37–3	135–117–33	233–51–1	260–24–1	79–105–31	116–134–35	172–96–17	224–55–6
	P value [a]	0.374	0.558	0.129	0.201	0.498	0.340	0.346	0.539	0.473	0.873	0.398	0.782	0.456

[a] *P* values of Pearson χ^2 tests.

Supplemental Table 10. Association between *ABCG2* SNPs and toxicity outcomes

SNP	Genotype	Toxicity	Toxicity grade (G3+G4/G0+G2)	*P* value [a]	OR (95% CI) [b]	*P* value [b]
rs12505410	A/A	Neutropenia	45/382	0.045	1.00 (ref)	
	A/C		48/345		1.19 (0.76–1.88)	0.442
	C/C		22/93		**2.29 (1.28–4.10)**	**0.005**
	C/C *vs* A/A+A/C		93/727	0.017	**2.09 (1.23–3.57)**	**0.007**
rs1871744	A/A	Anemia	20/443	0.079	1.00 (ref)	
	A/G		6/364		**0.37 (0.14–0.95)**	**0.039**
	G/G		3/108		0.57 (0.16–2.03)	0.387
	A/G+GG *vs* A/A		9/472	0.029	**0.42 (0.19–0.95)**	**0.038**
rs2231142	C/C	Neutropenia	56/377	0.036	1.00 (ref)	
	C/A		41/369		0.74 (0.48–1.15)	0.182
	A/A		18/74		1.51 (0.81–2.83)	0.199
	A/A *vs* C/C+A/C		97/746	0.025	1.73 (0.95–3.15)	0.073
rs2231138	A/A	Anemia	19/755	0.045	1.00 (ref)	
	A/G		10/153		**2.57 (1.12–5.89)**	**0.026**
	G/G		0/7		NA	NA
	A/G *vs* A/A+ G/G		19/762	0.013	**2.60 (1.13–5.94)**	**0.024**

[a] *P* values of Pearson χ^2 tests for overall distributions for SNPs genotypes between severe and mild toxicological phenotypes.
[b] Odds ratios (OR) and their 95% confidence intervals (CIs) and *P* values were calculated with unconditional logistic regression analysis, with adjustment of gender, age, smoking status, ECOG performance status, TNM status, histological types, and treatment regimen.

Supplemental Table 11. Association between objective response rate and overall survival

Stratification subgroup		Overall survival						
		n/N [a]		MST (m) [b]		Log-rank *P* [c]	HR (95%CI) [d]	*P* [d]
		nonresponse	response	nonresponse	response			
All		588/775	126/170	19.1	20.7	0.181	**0.82 (0.67–1.00)**	**0.050**
Gender	Male	428/548	92/125	17.7	21.7	0.062	**0.74 (0.59–0.94)**	**0.013**
	Female	160/227	34/45	24.4	20.2	0.701	1.20 (0.81–1.77)	0.357
Age	≤58	300/411	51/79	21.2	22.6	0.182	**0.73 (0.53–1.00)**	**0.049**
	>58	288/364	75/91	16.8	19.8	0.384	0.81 (0.62–1.07)	0.133
Smoking status	Smoker	351/449	77/103	17.1	21.7	**0.037**	**0.70 (0.54–0.91)**	**0.007**
	Nonsmoker	236/325	49/67	21.7	20.2	0.666	1.15 (0.83–1.58)	0.401
ECOG PS	0-1	528/699	117/156	19.1	21.3	0.280	0.82 (0.67–1.01)	0.067
	2	51/65	8/13	19.1	17.9	0.378	0.42 (0.18–1.01)	0.053
TNM stage	ⅢA	39/52	17/23	21.3	25.5	0.544	0.74 (0.39–1.40)	0.357
	ⅢB	167/220	44/55	18.7	20.7	0.325	**0.67 (0.47–0.95)**	**0.026**
	Ⅳ	378/498	65/92	19.4	20.0	0.597	0.89 (0.67–1.16)	0.381
Histologic type	AC	388/517	56/76	20.4	21.3	0.340	0.88 (0.66–1.17)	0.386
	SCC	117/153	43/57	13.7	23.0	**0.027**	**0.65 (0.45–0.94)**	**0.021**
Therapy regimens	Pt-navelbine	173/222	52/70	21.5	19.3	0.739	0.85 (0.62–1.18)	0.333
	Pt-gemcitabine	142/192	26/37	20.2	17.9	0.620	0.90 (0.58–1.41)	0.652
	Pt-paclitaxe	192/253	34/45	17.7	22.6	0.073	**0.58 (0.39–0.85)**	**0.005**
	Pt-docetaxel	54/70	9/11	17.7	23.3	0.808	0.80 (0.32–1.98)	0.621

[a] Numbers indicate the death event for NSCLC patients during the following-up time among all individuals in the same response or nonresponse group.
[b] MST: median survival time.
[c] Log-rank tests for association between survival and overall response.
[d] Hazard ratios (HR) and their 95% confidence intervals (CIs) and *P* values were calculated with by multivariate Cox proportional hazards regression with adjustment for covariates.

Supplemental Table 12. Association between objective response rate and progression free survival

Stratification subgroup		progression free survival						
		n/N [a]		MST (m) [b]		Log-rank *P* [c]	HR (95%CI) [d]	*P* [d]
		nonresponse	response	nonresponse	response			
All		461/715	94/159	7.7	12.4	**0.001**	**0.72 (0.58–0.91)**	**0.005**
Gender	Male	323/500	67/116	8.1	12.5	**0.003**	**0.69 (0.52–0.90)**	**0.007**
	Female	138/215	27/43	6.5	11.4	0.128	0.90 (0.58–1.39)	0.629
Age	≤58	254/382	40/73	7.1	12.5	**0.003**	**0.60 (0.42–0.85)**	**0.004**
	>58	207/333	54/86	9.0	11.7	0.107	0.81 (0.59–1.10)	0.179
Smoking status	Smoker	269/413	55/95	8.2	13.1	**0.003**	**0.65 (0.48–0.88)**	**0.005**
	Nonsmoker	191/301	39/64	7.1	10.5	0.115	0.80 (0.56–1.15)	0.228
ECOG PS	0-1	409/647	85/146	8.0	12.5	**0.004**	**0.75 (0.59–0.96)**	**0.021**
	2	44/59	9/13	3.7	8.8	0.111	0.58 (0.24–1.37)	0.211
TNM stage	ⅢA	25/43	13/21	9.6	13.1	0.652	0.56 (0.26–1.21)	0.141
	ⅢB	125/197	27/50	7.3	13.7	**0.015**	**0.56 (0.36–0.86)**	**0.008**
	Ⅳ	308/470	54/88	7.5	10.3	0.057	0.78 (0.58–1.06)	0.108
Histologic type	AC	316/484	43/73	7.8	12.6	**0.020**	0.75 (0.54–1.04)	0.085
	SCC	90/138	27/50	7.2	13.6	**0.005**	**0.62 (0.40–0.97)**	**0.035**
Therapy regimens	Pt-navelbine	112/220	36/69	13.8	12.5	0.313	0.75 (0.51–1.11)	0.151
	Pt-gemcitabine	119/173	24/33	9.4	8.8	0.917	1.07 (0.68–1.69)	0.773
	Pt-paclitaxe	166/232	24/40	5.7	14.6	**0.004**	**0.49 (0.31–0.78)**	**0.003**
	Pt-docetaxel	36/53	6/10	7.2	10.8	0.325	0.63 (0.20–1.99)	0.432

[a] Numbers indicate the event for NSCLC patients during the following-up time among all individuals in the same response or nonresponse group.
[b] MST: median survival time.
[c] Log-rank tests for association between PFS and overall response.
[d] Hazard ratios (HR) and their 95% confidence intervals (CIs) and *P* values were calculated with by multivariate Cox proportional hazards regression with adjustment for covariates.

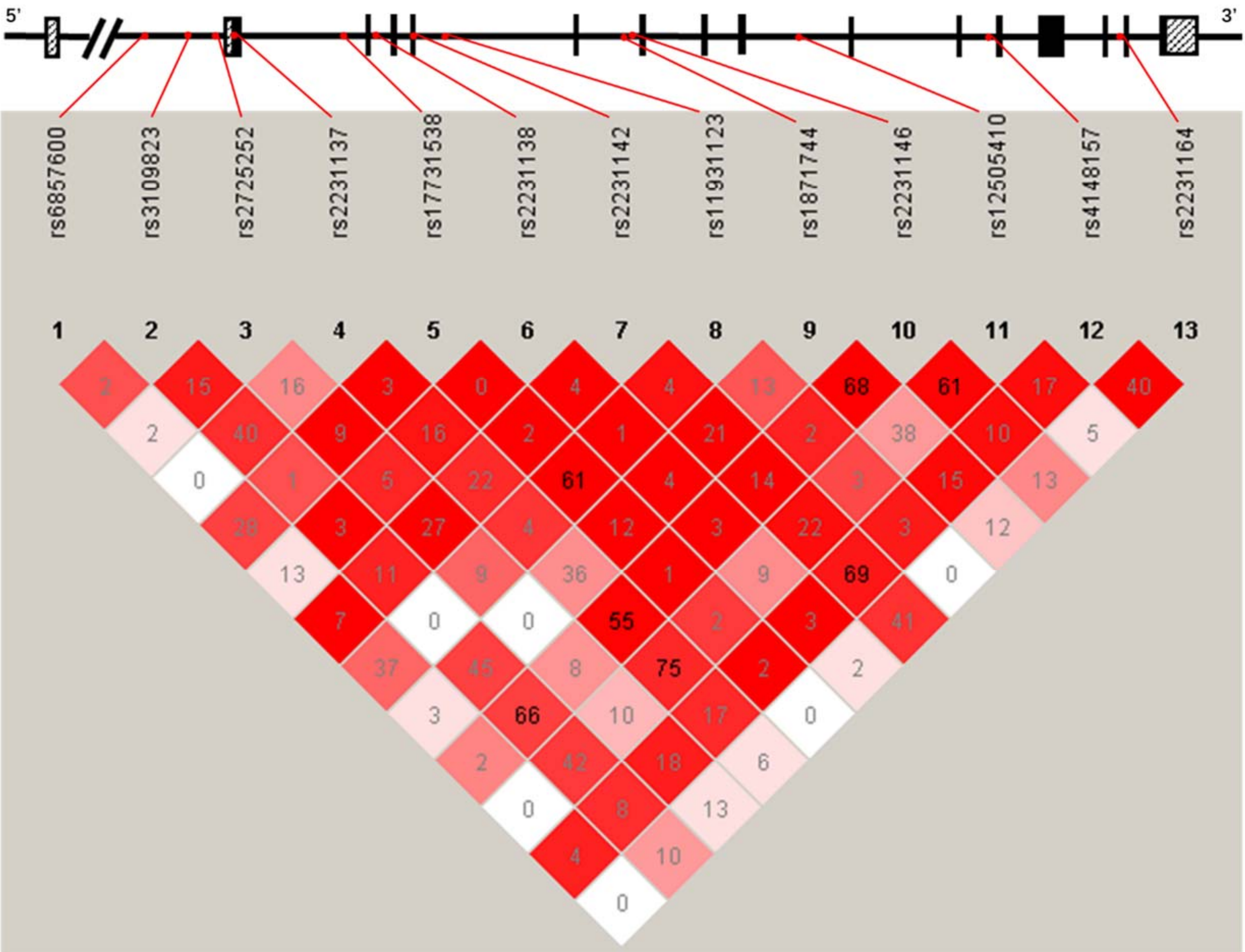

Supplemental Figure 1 Schematic structure of *ABCG2* gene and linkage disequilibrium of its candidate SNPs in the NSCLC cohort. The color intensity is proportional to D' and the percent numbers represent r^2.

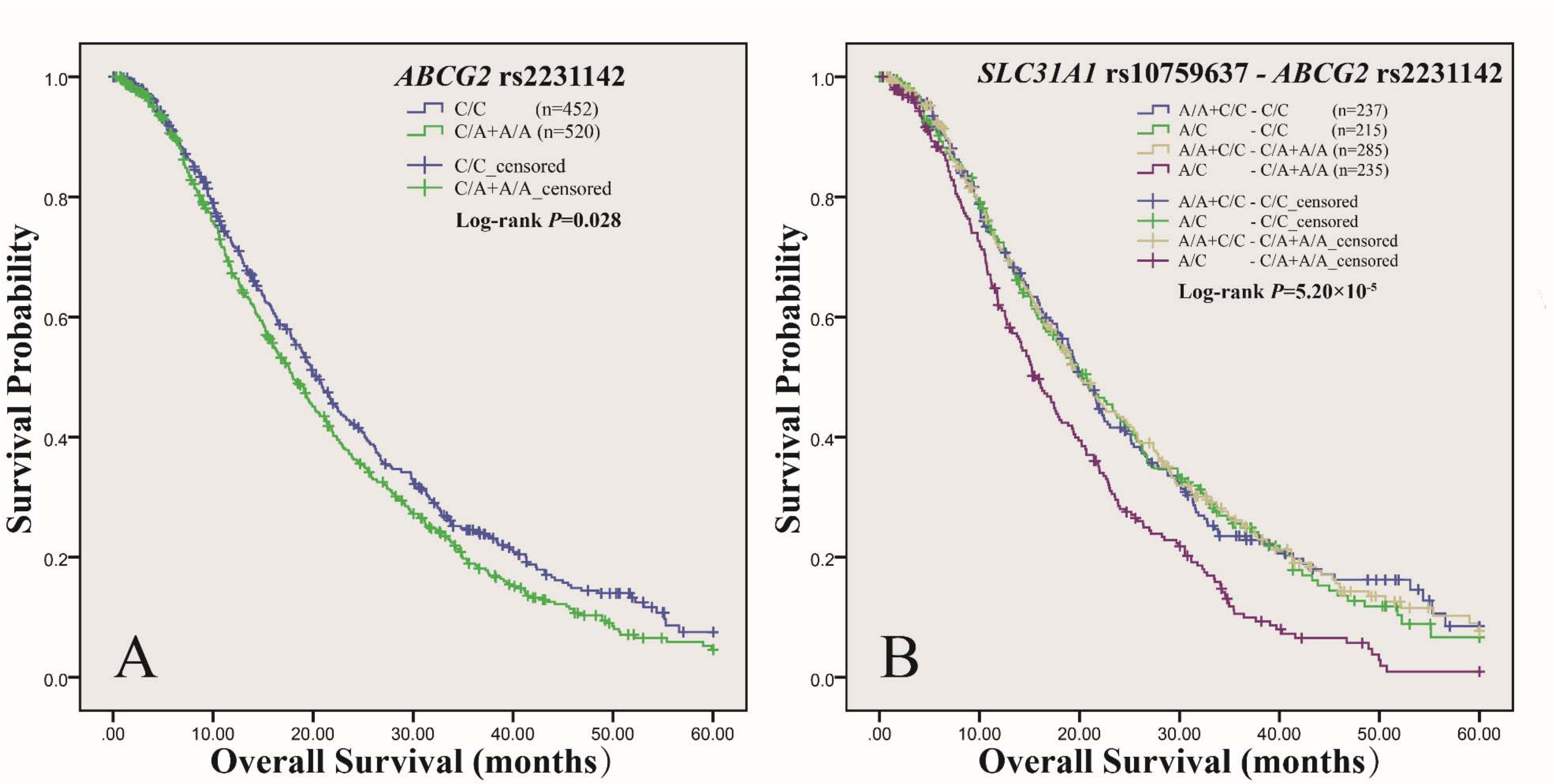

Supplemental Figure 2 Kaplan-Meier curve of estimated overall survival for the NSCLC cohort according to *ABCG2* rs2231142 and *SLC31A1* rs10759637 polymorphisms. The curves were plotted with SPSS software according to the genotypes of *ABCG2* rs2231142 (A) and the combined genotypes of *ABCG2* rs2231142 and *SLC31A1* rs10759637 (B). For rs2231142 C/A, the C/A+A/A genotypes group was compared to the wild C/C as reference in dominant model. For rs10759637 A/C, the A/C heterozygote was compared to the A/A+C/C homozygotes group as reference in under-dominant model.
